# Evolutionarily Conserved Linkage between Enzyme Fold, Flexibility, and Catalysis

**DOI:** 10.1371/journal.pbio.1001193

**Published:** 2011-11-08

**Authors:** Arvind Ramanathan, Pratul K. Agarwal

**Affiliations:** 1 Joint CMU–Pitt Program in Computational Biology, Carnegie Mellon University, Pittsburgh, Pennsylvania, United States of America; 2 Computational Biology Institute, and Computer Science and Mathematics Division, Oak Ridge National Laboratory, Oak Ridge, Tennessee, United States of America; Brandeis University, United States of America

## Abstract

Proteins are intrinsically flexible molecules. The role of internal motions in a protein's designated function is widely debated. The role of protein structure in enzyme catalysis is well established, and conservation of structural features provides vital clues to their role in function. Recently, it has been proposed that the protein function may involve multiple conformations: the observed deviations are not random thermodynamic fluctuations; rather, flexibility may be closely linked to protein function, including enzyme catalysis. We hypothesize that the argument of conservation of important structural features can also be extended to identification of protein flexibility in interconnection with enzyme function. Three classes of enzymes (prolyl-peptidyl isomerase, oxidoreductase, and nuclease) that catalyze diverse chemical reactions have been examined using detailed computational modeling. For each class, the identification and characterization of the internal protein motions *coupled to the chemical step in enzyme mechanisms* in multiple species show identical enzyme conformational fluctuations. In addition to the active-site residues, motions of protein surface loop regions (>10 Å away) are observed to be identical across species, and networks of conserved interactions/residues connect these highly flexible surface regions to the active-site residues that make direct contact with substrates. More interestingly, examination of reaction-coupled motions in non-homologous enzyme systems (with no structural or sequence similarity) that catalyze the same biochemical reaction shows motions that induce remarkably similar changes in the enzyme–substrate interactions during catalysis. The results indicate that the reaction-coupled flexibility is a conserved aspect of the enzyme molecular architecture. Protein motions in distal areas of homologous and non-homologous enzyme systems mediate similar changes in the active-site enzyme–substrate interactions, thereby impacting the mechanism of catalyzed chemistry. These results have implications for understanding the mechanism of allostery, and for protein engineering and drug design.

**Author's Summary**

Enzymes are nature's molecular machines that catalyze biochemical reactions with remarkable efficiency. Recent evidence suggests that enzyme function may involve not only direct structural interactions between the enzyme and its substrate, but also internal motions of the enzyme itself. Here, we describe a computational investigation of three classes of enzymes that catalyze completely different biochemical reactions. Remarkably, the mobile enzyme regions and the nature of these motions are the same across species ranging from single-celled organisms to complex life-forms. Also surprisingly, non-homologous enzymes that catalyze the same chemical reaction but do not share sequence or structural similarity reveal a similar impact of enzyme motions on their reaction mechanisms. Flexible enzyme regions are found to be connected by conserved networks of coupled interactions that connect surface regions to active-site residues. These networks may provide a mechanism for the solvent on an enzyme's surface to couple to the reaction catalyzed by the enzyme. These results have implications for understanding the mechanism of allostery (long-range effects), and for protein engineering and drug design.

## Introduction

Proteins are not static but rather are intrinsically flexible molecules. The relevance of conformational flexibility or multiple conformations of protein with small deviations from the native state to a protein's designated function is the subject of ongoing debate [1]–[7]. The role of protein structure in function such as enzyme catalysis is well established [8]. Techniques including X-ray crystallography and nuclear magnetic resonance (NMR) have been widely used to obtain information about the protein structure, thereby providing insights into the mechanism of function. The information obtained from these techniques reveals that the functioning protein is present in slightly different but related conformations, with some areas of the protein being more flexible than others. Given the success of structural effects in explaining many aspects of function, the observed fluctuations in structure have largely been ignored. More recently, however, it has been proposed that the protein function may involve multiple conformations, and that the observed deviations are not just inconsequential random thermodynamic fluctuations; rather, flexibility may be closely linked to protein function, including the catalytic efficiency of enzymes [2]–[3],[5]–[6],[9]–[15].

Internal protein motions span a wide range of length- and time-scales. The dynamical landscape of a protein and the associated energy landscape have been challenging to characterize, as the internal motions and the associated structural deviations occur over a broad range of time-scales [5],[16]. The fastest motions are harmonic vibrations of bonds and angles at femtoseconds (10^−15^ s) that have been linked to inducing changes in the crucial enzyme–substrate interactions [17]–[18]. The slower protein movements occurring at microseconds (and longer; >10^−6^ s) include global conformational fluctuations of large domains or of the entire protein, which include large displacements in surface loops as well as coordinated movement of β-strands and α-helices. There are also other movements that occur between these two extremes of time-scales. Experimental techniques including, but not limited to, NMR [3] and single-molecule experiments [19] have provided insights into the protein motions, at longer time-scales, in several enzyme systems including enzymes cyclophilin A (CypA) [5],[20]–[21] and dihydrofolate reductase (DHFR) [22]–[24]. The time-scales for the slow conformational changes and the chemical step catalyzed by these enzymes are similar, thus raising the question of whether they are interrelated [15].

Preliminary evidence has suggested the possibility that protein flexibility plays a promoting role in the biophysical mechanism of enzymes. Conformational flexibility of enzymes has been associated with substrate (and cofactor) binding and product release for some time now; however, the connection between flexibility and the chemical step still remains the subject of debate [2],[4]. In the enzyme human CypA, Kern and coworkers detected motions of several surface loop residues only in the presence of substrate [5],[20]–[21]. Agarwal and coworkers performed computational studies of CypA and identified a network of protein residues that influenced the reactive trajectories in the active-site [25]–[26]. For the hydride transfer catalyzed by DHFR, the groups of Benkovic, Wright, and Hammes, among others, have indicated the movement of surface loops Met20 and βF-βG in association with hydride transfer [23],[27]–[28]. Using computational methods, Hammes-Schiffer and coworkers have identified a network of coupled protein motions linked to enzyme function in DHFR [12],[29]. These networks formed by conserved residues both in and distal to the active-site have been implicated in promoting the catalytic step. The CypA and DHFR networks extend from flexible surface loop regions, which display high conformational flexibility, all the way to the active-site residues that directly participate in the catalysis. A number of other groups have also reported on the link between enzyme motions and catalysis [17],[30]–[33].

Several hypotheses have been proposed to explain the possible role of internal protein motions in enzyme catalysis. Pioneering work by Benkovic, Hammes-Schiffer, and coworkers has discovered that distal motions through a network of coupled motions assist hydride transfer catalyzed by DHFR [2],[34]. Wright and coworkers have characterized in detail the dynamic landscape of DHFR, where the ability of the enzyme to sample various conformations was shown to be closely linked to the progress along the enzyme cycle [3],[27],[35]. Kern and coworkers have proposed that the catalysis promoting dynamics is an intrinsic property of enzymes [20],[36]. Collectively, an integrated view of enzyme structure, flexibility, and function is emerging based on the hypothesized role of protein motions in enzyme mechanisms [15]. Along with the structural interactions, internal motions at fast time-scales control the chemical environment of the active-site favoring the catalytic step to proceed to the product state [26]. It has been hypothesized that the solvent is thermodynamically and energetically coupled to the flexible surface loops, which eventually transfer the kinetic energy to the active-site through the conserved network interactions [15]. Evidence from computational studies as well as from Mössbauer and neutron scattering studies supports the hypothesis that thermodynamical fluctuations in the hydration-shell and bulk solvent control the behavior of reactive trajectories [26],[37]. The thermodynamical conformational fluctuations in the networks alter the enzyme–substrate interactions so that more reaction trajectories cross the transition state barrier to reach the product state successfully. Overall, the role of protein motions in promoting the substrate turnover step lies in facilitating the attainment of the transition state and by enabling more successful reactive trajectories.

Factors others than protein flexibility also play an important role in enzyme catalysis. Warshel and coworkers have proposed that the electrostatic effect and the effect of solvent reorganization make important contributions to many enzyme mechanisms [38]–[39]. Further, Bruice and coworkers have proposed the *near-attack-conformation* theory, which suggests that enzyme active-sites are set up to preferentially bind to the substrate conformations that are in the vicinity of TS [40]. The extent to which these different factors, including flexibility, impact enzyme catalysis still remains an unsolved mystery.

Conservation of structural features across species has provided vital clues to their role in protein function. For example, information using sequence profiles of several enzyme super-families including dehydrogenases, enolases, and amidohydrolases/phospotriesterases has led to the identification of conserved structural features associated with targeted chemistry [41]–[42]. In particular, it has been argued that the enzyme active-site residues are optimally arranged to provide a complementary environment to the transition state to allow for its stabilization [43]–[44]. (Some residues are also conserved for their role in folding and protein stability.) The overall enzyme shape or the *enzyme fold* has been suggested as a scaffold that serves to correctly position the conserved active-site residues. This notion has led to the *structure encodes function* paradigm, with a number of theories strongly emphasizing the structural interactions between the enzyme and the substrate.

We hypothesize that the argument of conservation of important structural features can also be extended to identification of protein flexibility in connection with enzyme catalysis. Similar to individual residues and motifs that are conserved in enzymes, for their structural role, we suggest that the chemistry promoting flexible regions of enzymes and their motions are also conserved as a part of the enzyme fold. Previous studies have already reported a connection between substrate (and cofactor) binding/release and the intrinsic dynamics of the enzyme fold [45]–[46] and conservation of dynamics across enzyme families and super-families [47]–[48]; in this study the focus is on how the protein flexibility is linked to the chemical step during the enzyme cycle (after substrate/cofactor binding and before the removal of the product). It is important to note that for the enzyme systems selected for this study, *the investigated chemical step is the rate-limiting step*. Therefore, the focus of our investigations is to identify the enzyme conformational fluctuations that are correlated with the critical step involving the mechanism that limits the overall rate of enzyme function. This approach for identification of enzyme flexibility closely coupled to the rate-limiting step provides vital insights into the connection between enzyme motions and function.

In this report, we describe our computational investigations of protein flexibility linked to enzyme catalysis to test the connection between the enzyme's fold, conformational flexibility, and function, as well as its conservation over evolution. Three well-characterized enzymes catalyzing different types of chemical reactions with distinct folds and reaction mechanisms are investigated: CypA, a member of the peptidyl-prolyl isomerase family, which catalyzes *cis/trans* isomerization of peptide bonds; DHFR, a member of oxidoreductase family, catalyzing hydride transfer; and ribonuclease A (RNaseA), a member of nuclease family that catalyzes hydrolysis of single-stranded RNA. For each enzyme fold, computational investigations of enzyme structures from several species with different sequences have been performed. Slow conformational fluctuations at the time-scale of the reaction and spanning the entire enzyme structure have been characterized.

A critical test for our hypothesis is provided by comparison of reaction-coupled enzyme flexibility in non-homologous enzymes. Identification of motions and flexibility linked to mechanism in enzymes that catalyze the same chemistry, but have little or no similarity in sequence and have different molecular architectures or structure, provides vital insights into the connection between enzyme structure, flexibility, and function. The isomerization of peptide bonds by non-homologous peptidyl-prolyl enzymes CypA and Pin1 has been characterized and compared. Similarly hydride transfer catalyzed by two non-homologous DHFRs (the well-known chromosomally encoded DHFR and a plasmid encoded type II DHFR) have also been compared. Further, an example of the negative consequence of truncation of a catalytically relevant flexible surface loop in RNaseA is also discussed. The results indicate the presence of protein motions in distal areas of the dissimilar enzyme folds that mediate similar changes in the active-site enzyme–substrate interactions, thereby impacting reaction mechanisms. Our studies have led to the discovery that in homologous as well as non-homologous enzyme systems the protein motions coupled to the reaction mechanisms are conserved features of the enzyme fold. Similar to the insights provided by conserved residues and structural motifs, future investigations of the identified dynamical regions will provide a more detailed understanding of the role of internal protein motions in enzyme catalysis.

## Results

### Peptidyl-Prolyl Isomerase

CypA is a peptidyl-prolyl isomerase (PPIase) catalyzing the *cis/trans* isomerization of peptide bonds in small peptides and proteins [49]. The molecular architecture of human CypA consists of an eight-strand anti-parallel β-barrel with the active-site located on one face. The active-site consists of a hydrophobic pocket with conserved residue F113, where the target proline residue is held fixed during the reaction [25],[50]. There are also conserved residues that form hydrogen bonds with the substrate backbone, with R55 playing an important catalytic role [51]. The enzyme structure shows the presence of several flexible surface loop regions distal to the active-site, with conserved residues located over 15 Å from the active-site. NMR spin-relaxation studies performed by Kern and coworkers indicated a link between the motions of several residues and the substrate turnover step, and also indicated that the rate of enzyme conformational changes coincides with the rate-limiting step of substrate turnover (about a hundred microseconds) [20],[36]. In particular, motions in loop regions neighboring the active-site residues R55, N102, and A103, as well as in distal residues L82 and S110, were observed only in the presence of substrate. Additionally, regions away from the active-site with residues T68 and G72 indicated enhanced motions in the presence of substrate.

Previous computational modeling of human CypA performed by our group has led to the discovery of a network of protein vibrations promoting the catalytic step [25]. Slow conformational fluctuations spanning the entire enzyme and coupled to the isomerization reaction were identified. In particular, three slow conformational modes coupled to the reaction were characterized for their connection with the biophysical mechanism. The coupling to the reaction is defined as the degree of variation in the amide bond dihedral angle (see Materials and Methods section for details). A network of coupled vibrations was discovered, which is formed by the connection of flexible surface loop regions to the active-site, and includes residues R55, N102, A103, G104, T107, N108, and G109. It was hypothesized that the movement in flexible surface loops is driven by solvent thermodynamical fluctuations, which in turn through the network linkages makes an impact on the reaction by regulating the crucial active-site interactions so that more trajectories become productive [26]. NMR studies had confirmed the presence and dynamical movements of critical parts of the network coupled to the substrate turnover [36].

#### Quantitative comparison of reaction-coupled CypA flexibility across multiple species

**Figure 1:**
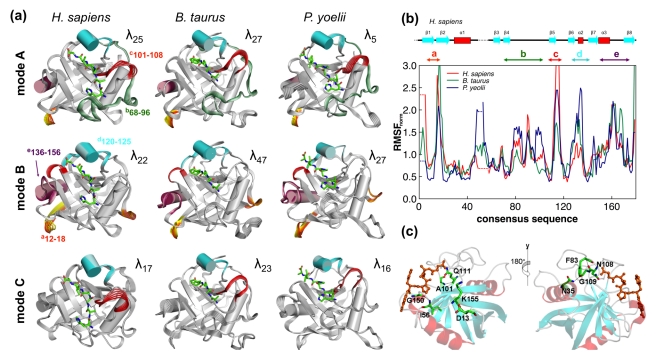
Conservation of reaction-coupled flexibility in enzyme CypA across three different species. (a) Top three slowest modes coupled to the *cis/trans* isomerization reaction show large fluctuations in identical regions (near and away from the active-site). Multiple snapshots are shown to indicate movements along the modes, and the regions with high flexibility are shown in color. The depicted modes are the ones showing largest coupling to the reaction and are ranked differently from the slowest modes for the entire enzyme–substrate complex (λ_N_ indicates the rank of each mode based on the eigenvalue provided by quasi-harmonic analysis, with λ_1_ corresponding to slowest mode). (b) Enzyme backbone flexibility depicted as root mean square fluctuations (RMSF); computed by aggregating the C_α_ displacement magnitude in the top 10 modes coupled to the reaction. For comparison, consensus sequence has been used and RMSF has been normalized by dividing by the average C_α_ flexibility of all residues in the enzyme (see Text S2 for the consensus sequences). Also, the regions marked as “a–e” correspond to the colored regions in panel (a) that show the largest displacements in the modes. (c) Conservation of the network interactions connecting the flexible regions as a part of the CypA fold (only human CypA is shown; however, these interactions are conserved in human cyclophilin B, CypA from *Brugia malayi*, *B. taurus*, and *E. coli* as well). See Text S1 and Movies M1–M9 for more details.

Characterization of the reaction-coupled conformational flexibility in CypA from humans, *Bos taurus*, and *Plasmodeum yoelii* has revealed remarkably identical flexibility (see Figure 1). To obtain quantitative understanding of reaction-coupled enzyme flexibility across species, we characterized the top 10 modes coupled to the PPIase reaction (see Tables 1–2). As Table 1 depicts, the degree of coupling to the reaction is quantitatively similar in these different species. Note that these three modes are not the slowest modes of the enzyme, but the *slowest modes that show the largest coupling to the reaction*. The slowest modes were ranked by coupling to the reaction and only the top three modes were characterized in detail. As the associated number λ_n_ (ranking by eigenvalue) indicates, the slowest mode of the entire enzyme complex does not always correspond to the slowest mode with largest coupling to the reaction. For quantitative estimates of similarity between these modes, the sub-space overlap metric developed by Hess was used [52]. As Table 2 shows, the top 10 reaction-coupled modes in CypA show 66%–69% similarity across species even though they share only an average of 58% sequence similarity.

**Table 1:** Degree of coupling of the slow conformational modes to the *cis/trans* isomerization catalyzed by CypA.

Species	Mode A	Mode B	Mode C
*H. sapiens*	2.545	2.083	2.028
*B. Taurus*	2.261	2.082	1.454
*P. falciparum*	2.121	1.936	1.863

See text and Figure 8 for the methodology used for coupling calculations. To allow comparison of the coupling between species, values were normalized by the average coupling in slowest 50 modes computed from quasi-harmonic analysis (QHA).

**Table 2:** Similarity of reaction-coupled modes in CypA.

Species	*H. sapiens*	*P. falciparum*
*B. taurus*	0.691	0.665
*P. falciparum*	0.660	

The subspace overlap was computed using Hess' metric for the top 10 modes coupled to the *cis/trans* isomerization reaction catalyzed by CypA.

Further, not only the collective sub-space overlap from the top 10 modes but the top three slowest protein vibrational modes coupled to the *cis/trans* isomerization are individually conserved over evolution (see Figure 1a). These three reaction-promoting modes show identical flexibility in the distant areas of the enzyme, even though the protein structures are from different species (see Figure 1b and Movies S1–S3). The correlation of the atomic fluctuations in these three reaction-coupled modes is more than 75%, indicating that the protein regions showing large displacements within these reaction-coupled modes, as well as their direction and amplitudes, are identical (highlighted in Figure 1b as regions *a–e*). To rule out the possibility of biasing the active-site dynamics, three different substrates were used for these simulations. The protein regions showing large displacements within these modes have also indicated large fluctuations as measured by N15 spin-relaxation experiments, in the presence of substrate [20],[36].

Overall, the characterization of intrinsic CypA flexibility in the structures from three species shows the presence of regions of similar dynamical fluctuations, as indicated by a dynamic clustering analysis of protein regions (see Figure S1). The protein residues separate into six clusters based on their characteristic flexibility over the course of isomerization; these include the β-sheet separating into two rigid clusters, two helices and associated loops forming two additional clusters, and the surface loop regions forming two flexible clusters. Dynamical cross-correlation and structural analyses of the PPIase fold (Figures S2–S3) indicate the regions showing reaction-coupled flexibility are connected by a network of hydrogen bonds.

#### Networks of coupled interactions promoting catalysis in CypA

The characterization of CypA fold provides mechanistic insights into the role of conserved flexibility in promoting the isomerization reaction. During the course of the reaction, the target substrate proline ring is held fixed in the rigid active-site. The motions of the enzyme residues A101–N102–A103 and nearby loop 105–108, as well as R55 in human CypA, alter the crucial enzyme–substrate interactions during the reaction. Additionally, motions of F60 in the active-site and the associated loop region 57–60 also make important contributions to the reaction mechanism. In *B. taurus*, the equivalent residues A121–N122–A123 and the loop 125–127 also show large movement during enzyme catalysis, with conserved active-site residues R75 and F80 also displaying similar motions. In *P. yoelii*, the residues A143–N144–S145 and the loop 147–149 also display large movements during the course of the reaction, with active-site residues R97 and F102 controlling the crucial enzyme–substrate interactions. More interestingly, several regions distal to the active-site also display a similar type of dynamical motions in the slowest modes coupled to the isomerization reaction. These regions highlighted in Figure 1 include the highly flexible surface loops (e.g., the region 82–88 in human CypA).

Note, the residues and interactions forming these networks as well as their motions are conserved over evolution (Figure 1c). These interactions originate in the highly flexible surface loop regions on opposite sides of the protein (F83N–N108O, N35N_δ2_–G109O, and D13N–K155O in human CypA) and pass through internal regions (A101N–Q111O and I56N–G156O) to eventually connect to the residues involved in structural contacts with the substrate (R55, F60, N102, and A103). It is interesting to note that even though the exact residues are not conserved, the linkage at the particular location is preserved to keep the network intact. The presence of regions with similar dynamical characteristics in distal areas of the protein (away from the active-site), the similarity of clusters of residues showing coupled motions, and the conservation of linkages connecting the flexible surface loop regions all the way to the active-site regions form important features of the PPIase (CypA) enzyme fold. Preliminary indications are that these networks are pathways of energy connectivity between the active-site residues and the surface regions (therefore, the surrounding solvent) [26].

#### Non-homologous PPIase Pin1

Pin1 is also a PPIase that catalyzes the isomerization of the peptidyl-prolyl bonds [49],[53]. Although Pin1 catalyzes the same reaction as CypA, a difference between the two enzymes is that Pin1 is preferential to the isomerization of phosphorylated substrates (pSer–Pro or pThr–Pro motifs). The Pin1 structural fold shows no similarity to CypA; it consists of an N-terminal WW domain (residues 1–39) and a C-terminal PPIase domain (residues 45–163). NMR studies indicate that the intrinsic flexibility of CypA and the PPIase domain of Pin1 are “primed” for catalysis, indicating that the free enzyme samples the motions that impact enzyme mechanisms [54]. The intrinsic dynamics of these enzymes show correlated motions (between different enzyme residues) at the microsecond-to-millisecond time-scale.

The structural dissimilarity between CypA and Pin1 folds poses a challenge for a direct comparison of the impact of reaction-coupled flexibility linked to PPIase mechanism. Hence, an alternate strategy was used to gain insights into the similarity between the flexibility linked to the reaction mechanisms in two folds. A view of Pin1 and CypA active-site environment indicates that the substrate prolyl ring is held in a hydrophobic pocket (surrounded by F113 in human CypA and L122 in Pin1) and the loops in proximity to the active-site show significant flexibility coupled to the reaction pathway. In particular, the Pin1 active-site residue R68 is connected to surface regions of large flexibility (63–82), similar to R55 in CypA. Note our past investigations of the CypA fold have revealed that several residues (both proximal and distal to the active-site) play an important role in altering the active-site environment through a series of coordinated interactions [25]. Therefore, we examined if any flexible loops and interaction in Pin1 induce similar effects on the PPIase mechanism.

**Figure 2:**
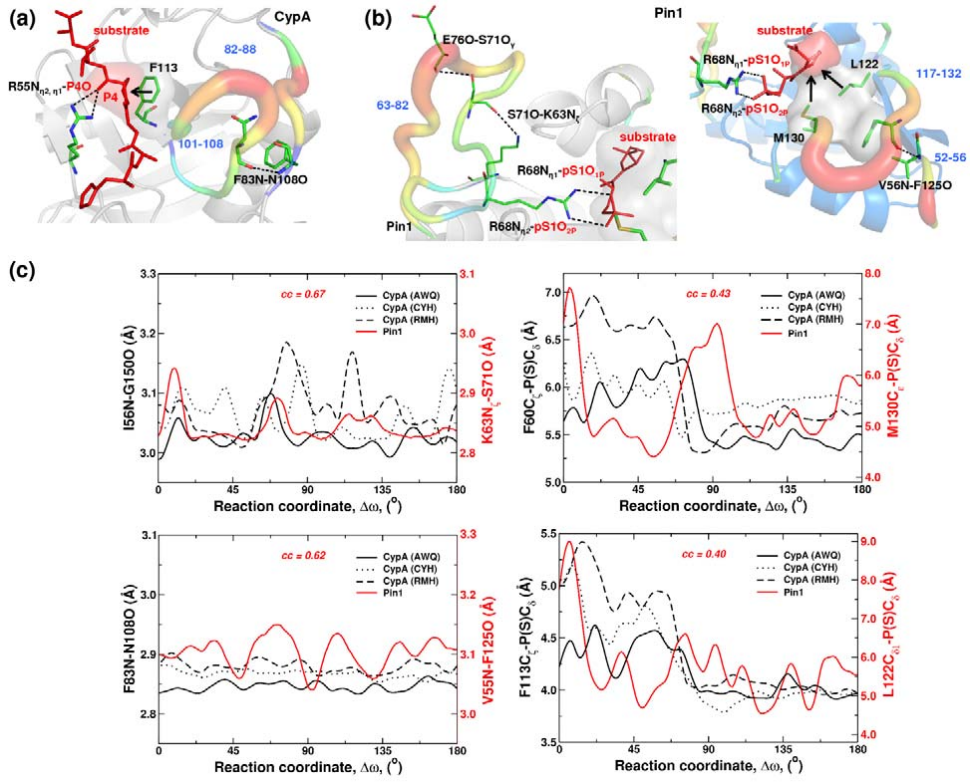
Comparison of reaction-coupled flexibility in non-homologous PPIases. PPIases CypA and Pin1 with dissimilar structural folds catalyze the isomerization of peptidyl-prolyl peptide bonds. (a) In CypA, the flexible surface regions 82–88 and 102–108, connected by F83N–N108O, impact the residue substrate preceding the target proline (F113 as well as N102 and A103) and region flexible region 57–60 is connected to substrate network residue R55. (b) In Pin1 flexible surface regions 52–56 and 117–132 are interconnected by V56N–F125O relay motions into the active-site through hydrophobic residues L122 and M130. On the other face of Pin1 the flexible surface regions 63–82 are also connected by network hydrogen bond to R68 that forms hydrophilic interactions with substrate similar to R55 of CypA. (c) Similarity in the behavior of distal (I56N-G150O/K63N_ζ_-S71O and F83N-N108O/V55N-F125O) as well as active-site interactions (F60C_ζ_-P(S)C_δ_/M130C_ε_-P(S)C_δ_ and F113C_ζ_-P(S)C_δ_/L122C_δ1_-P(S)C_δ_) for CypA and Pin1. Equivalent network surface network hydrogen bonds impact the enzyme–substrate interactions in the active-site. Note, *cc* values indicate correlation coefficients between CypA (AWQ) and Pin1 interactions calculated over the course of reaction profile. See ref. [25] for further details.

Computational modeling of the Pin1 PPIase reaction provides atomic-level insights into the reaction-coupled flexibility (see Figure S4 and Text S1 for details). The active-site residues that form direct contacts with the bound substrate are interconnected to flexible surface loop regions (see Figure 2), in a manner similar to the case of CypA. The location and the role of reaction-coupled flexibility in mediating enzyme–substrate interactions in active-site of CypA and in Pin1 are remarkably similar, even though there is no sequence or structural similarity. A network of hydrogen bond interactions (formed by E76O-S71O_γ_ and S71O-K63N_ζ_) extends from the surface region and connects this flexible surface loop into the more rigid active-site residues, eventually interacting with the substrate. Further, to stabilize the hydrophobic pocket in the active-site for the substrate proline, M130 and L122 provide important interactions, similar to the roles of F60 and F113 in CypA. Note that this Pin1 hydrophobic pocket is also surrounded by flexible regions 52–56 and 117–132 that show large displacements and are interconnected through hydrogen bond V56N–F125O, similar to the case of CypA where the flexible region 101–108 and 82–88 are connected by hydrogen bond F83N-N108O. A comparison of these active-site and distal network interactions over the course of catalyzed isomerization reaction reveals a remarkably similar behavior.

Quantitative comparison of these interactions in the two enzyme shows correlation coefficients ranging from 0.40–0.67 (see Figure 2c) even though different types of residues are involved, and they are located far away from the active-site in dissimilar enzyme folds. The motions in Pin1 network residues mediate changes in the active-site chemical environment to facilitate the isomerization of the peptide bond, very similar to changes mediated by the CypA network residues [25].

Pin1 residues K63, R68, and S71 in the substrate-binding loop (63–82) as well as L122 and M130 in the flexible loop 117–132, which form part of the network, are conserved across multiple species [55]. Further, directed evolution experiments indicate that several of the residues located in these two loops (63–82 and 117–132) are known to significantly affect the catalytic process in this enzyme [55]. The surface loops that form part of the network (and show the presence of residues with long side-chains) undergo significant conformational exchange during the Pin1 catalytic cycle, as evidenced by NMR experiments [54].

### Oxidoreductase

DHFR catalyzes the reduction of 7,8-dihydrofolate (DHF) to 5,6,7,8-tetrahydrofolate using nicotinamide adenine dinucleotide phosphate (NADPH) as a cofactor. Chromosomally encoded DHFR belongs to a family of proteins sharing the nucleotide binding Rossmann fold [56], characterized by a central core formed β-sheet surrounded by α-helices. Previously, a network of coupled motions promoting hydride transfer in DHFR had been identified using detailed theoretical and computational modeling [12]. Similar to the network in CypA, this network is also formed by surface residues present on the flexible loop regions (particularly the βF–βG and the Met20 loop) interacting with other conserved residues all the way to the active-site. The detailed characterization of correlated motions of various residues and this network has led to the identification of a chain of residues as dynamical contributors to hydride transfer reaction [11]–[12],[57]–[58]. Long time-scale fluctuations (around milliseconds) in these loop areas have been linked to the mechanism of the hydride transfer. This is particularly intriguing because at pH >8.4, *hydride transfer is the rate-limiting step in the entire catalytic cycle* [59].

#### Quantitative comparison of reaction-coupled DHFR flexibility across multiple species

**Figure 3:**
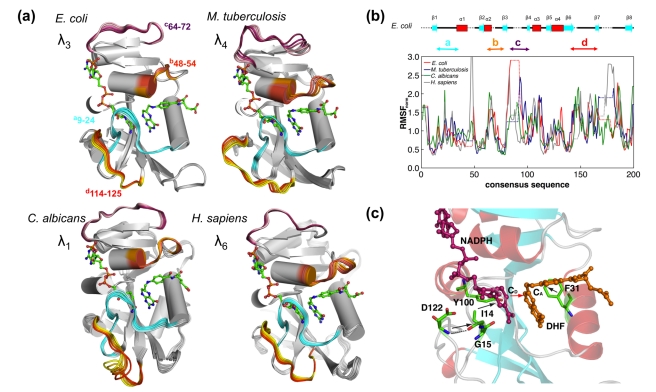
Conservation of reaction-coupled flexibility in enzyme DHFR across four species. (a) Slowest mode coupled to the hydride transfer catalyzed by these chromosomally encoded DHFRs shows large fluctuations in the same regions (near and away from the active-site) of the enzyme from four species. (b) Enzyme backbone flexibility depicted as normalized RMSF. (c) Conservation of the network interactions (black arrows) connecting the flexible regions as a part of the DHFR fold (only *E. coli* DHFR is shown). The red arrow indicates the catalyzed hydride transfer between C_D_ and C_A_. The modes are depicted/colored and the RMSF is normalized similarly to the CypA results. See legend of Figure 1 for further details.

Conformational flexibility linked to the hydride transfer catalyzed by DHFR from *Escherichia coli*, *Mycobacterium tuberculosis*, *Candida albicans*, and humans shows considerable movements in the surface loop regions. Table 3 shows quantitative similarity in the degree of coupling to the hydride transfer for the top most reaction-coupled modes across the four species. The coupling to the reaction is defined as the dot product between displacement vector of hydride and the vector between donor and acceptor carbon atoms (see Materials and Methods section for details). Similar to CypA, DHFR from different species also shows a complete similarity of motions in the equivalent regions (see Figure 3). Note these motions correspond to the time-scale of hydride transfer. Top 10 modes reaction-coupled show similar flexibility in equivalent regions (highlighted as regions in *a–d* in Figure 3). A quantitative comparison of the top 10 reaction-coupled modes shows 53%–61% similarity even when the sequences are only 30% similar on average (see Table 4). In addition to the sub-space overlap, the individual vibrational modes coupled to the hydride transfer reaction are also conserved across species (see Figure 3, and Movies S4–S6).

**Table 3:** Degree of coupling of the slow conformational modes to the hydride transfer catalyzed by DHFR.

Species	Mode A	Mode B	Mode C
*E. coli*	4.634	3.458	2.520
*M. tuberculosis*	4.329	3.328	3.007
*C. albicans*	3.699	3.291	2.673
*H. sapiens*	3.816	3.472	2.443

See text and Figure 8 for the methodology used for coupling calculations. To allow comparison of the coupling between species, values were normalized by the average coupling in slowest 50 modes computed from QHA.

**Table 4:** Similarity of reaction-coupled modes in DHFR.

Species	*C. elegans*	*M. tuberculosis*	*H. sapiens*
*E. coli*	0.534	0.543	0.613
*C. elegans*		0.531	0.541
*M. tuberculosis*			0.549

The subspace overlap was computed using Hess' metric for the top 10 modes coupled to the hydride transfer catalyzed by DHFR.

Overall, the characterization of the reaction-coupled flexibility shows strong correlated motions in DHFR fold in spite of sharing low sequence similarity (see Figures S5–S6). The dynamic clustering method indicates that over the course of hydride transfer reaction the dynamical motions of the β-strands separate into two clusters (see Figure S5). Three additional clusters are formed by helices and the loops. The most flexible cluster is formed by the Met20 and βF–βG loops near the cofactor nicotinamide ring, the substrate binding pocket, and the adenosine binding domain. This clustering was the same across all of the four species investigated, implying that the dynamical coupling between different parts of the DHFR fold is conserved.

#### Networks of coupled interactions promoting catalysis in DHFR

The most characteristic feature of the slowest vibrational modes is the high degree of activity in the surface loop Met20 and βF–βG loops as well as the substrate binding pocket, which is close to the substrate DHF's para-aminobenzoylglutamate (p-ABG) tail. These regions have an impact on the reaction by positioning the nicotinamide ring of the cofactor in close proximity to the substrate ring, and by decreasing the distance between donor–acceptor carbons (C_D_–C_A_) for hydride transfer. These regions contain the residues that form the network of coupled protein motions including the Y100, I14, and F31 in *E. coli* DHFR. Additionally, residue R57 shows concerted movement with the DHF tail. Structural analysis indicated that these residues and the interactions are also conserved over evolution and display identical motions along the reaction pathway in *M. tuberculosis* (Y100, I14, F31, R60), *C. albicans* (Y118, I19, F36, R72), and the human (Y121, I16, F34, R70) enzyme.

The link between the DHFR fold and the reaction-coupled flexibility is similar to that in CypA. The clusters of flexible surface loops are connected to the active-site residues through the preserved linkages (see Figure 3c and Figures S6–S7). Particularly, the surface hydrogen bond D122–G15 in *E. coli*, D126–G15 in *M. tuberculosis*, D146–G20 in *C. albicans*, and D145–G15 in the human enzyme is conserved. Therefore, these regions also form a characteristic feature of the enzyme fold with implications for catalysis. Note that the importance of network and nearby residues to the enzyme mechanism has been confirmed by mutation studies [58]. Further, the Met20 loop of *E. coli* DHFR, in particular, is known to exist in two conformations, occluded and closed, and has been implicated in the catalytic step. Single-molecule experiments have also suggested the concerted movement of this loop with the hydride transfer [28].

#### Non-homologous R67 DHFR

**Figure 4:**
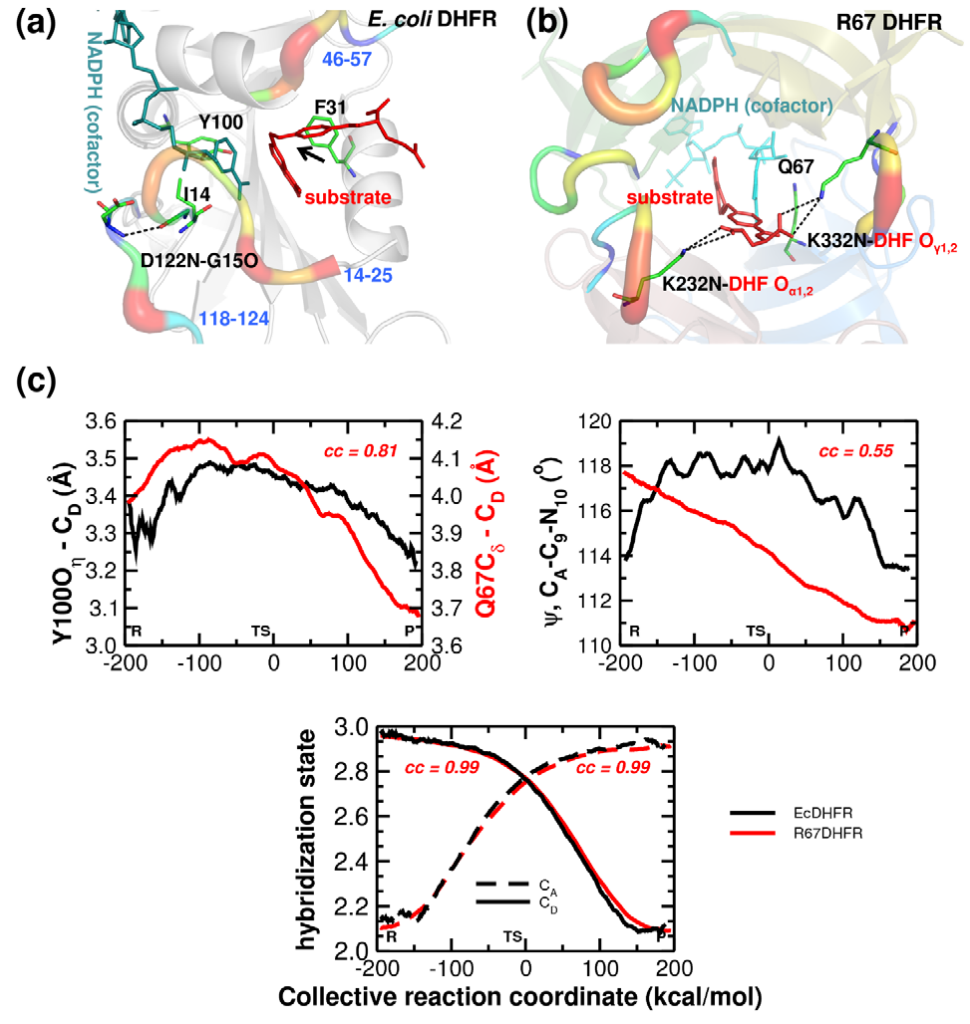
Comparison of reaction-coupled flexibility in non-homologous DHFRs. (a) Hydride transfer between cofactor NADPH and substrate DHF catalyzed by *E. coli* DHFR and R67 DHFR with dissimilar enzyme folds. In *E. coli* DHFR residues D122, G15, I14, and Y100 provide promoting motion to the cofactor nicotinamide ring and F31 motions alters the chemical environment on the reactant [12]. In R67 DHFR the Q67 provides the promoting motion to the nicotinamide ring and the movement of substrate tail in open pore, controlled by hydrophilic interactions between residues K32 from two different sub-units (labeled as K232 and K332) and α/γ carboxylate groups of the substrate p-ABG tail. Note the four monomers in R67 DHFR are colored differently and the hydrophilic interaction between the cofactor tail and the K232 and K332 are shown. (b) The network residue Y100 in *E. coli* DHFR and Q67 in R67DHFR are positioned behind the nicotinamide ring and provide similar promoting motions to C_D_. The puckering of the pteridine ring of the DHF substrate at the C_A_ is also a contributor to the reaction coordinate. The change in DHF-tail angle (ψ, C_A_–C_9_–N_10_) from *sp2* (∼120°) to *sp3* (∼109°) state of hybridization is induced by different features of the enzyme folds. The overall effect on the reaction center chemical environment is the same as indicated by the state of hybridization of the C_D_ and C_A_. Note, *cc* values indicate correlation coefficients between the quantities from the two folds calculated over the course of reaction profile. See ref. [62] for further details.

This plasmid-encoded type II DHFR also catalyzes the transfer of hydride from cofactor NADPH to substrate DHF; however, it shows neither sequence nor structural homology with the chromosomal DHFRs [60]–[61]. This plasmid encoded R67 DHFR was discovered due to its ability to confer trimethoprim resistance upon host bacteria. The structure of chromosomally encoded DHFRs (discussed above) displays the Rossmann fold [56], which is a characteristic feature of many dinucleotide binding proteins. The structure of R67 DHFR shows an SH3-like domain and consists of a homo-tetramer (each subunit 78 amino acids in length) with the active-site located in the middle of a pore that is mostly accessible by bulk water [61]. R67 DHFR shows a number of characteristics of a primitive enzyme including promiscuity in binding of substrate/cofactor, formation of non-productive complexes, and the absence of a conserved acid in its active-site [60]. Even though the structures of DHFR and R67 DHFR show no similarity, computational and experimental investigations have revealed interesting similarities in the reaction-coupled flexibility for the two enzyme folds (see Figure 4) [62]. The active-sites show stacking between the nicotinamide (of cofactor NADPH) and pteridine (of the substrate DHF) rings enabled by a number of enzyme residues.

More interestingly, there are similarities with regards to the relative motions—both in the active-site and in distal regions of the enzyme— that alter the chemical environment, making it suitable for catalysis to occur (see Figure 4c). In particular, puckering of the NADPH ring and a change in the DHF-tail angle coupled with the hydride transfer are observed in both enzyme systems. In *E. coli* DHFR, it is suggested that the nicotinamide ring puckering motion has been suggested to be induced by a network of coupled motions originating from D122 on the surface and terminating in Y100 in the active-site, positioned behind the C_D_. In R67 DHFR, the Q67 side-chain appears to provide similar motions to the C_D_, also positioned behind the cofactor ring. Puckering of the nicotinamide ring has been suggested as a contributor to the reaction coordinate in the dinucleotide (NADPH/NADH) binding enzyme [63]. The correlation coefficients for the measured quantities range between 0.55 and 0.99 (see Figure 4c), indicating a remarkably similar impact of protein flexibility on the mechanism in the two DHFRs.

The most interesting difference in the enzyme mechanism appears to impact the chemical environment at the C_A_. In *E. coli* DHFR, it has been suggested that the motions of the F31 side chain provide promoting motions that alter the DHF-tail angle, thereby making the C_A_ more suitable for the incoming hydride. In R67 DHFR, computational studies predict that the same change in the chemical environment results from the substrate's p-ABG tail movements [62]. Sampling of the DHF-tail angle is made possible by the high degree of flexibility in the tail located at the edge of the pore surrounded by bulk solvent. The two extreme states for the conformations are ion-pairs between the α/γ-carboxylate groups of DHF interacting with symmetry-related K32 residues from two different subunits (labeled as K232 and K332). A loss of these ion-pair interactions (located >13 Å from the center of the pore) in K32M mutants leads to altered enzyme kinetics. The tail movement at the edge of the active-site, coupled with the fixed position of the pteridine ring in the center of the pore, leads to puckering of the pteridine ring and promotes transition state formation. Overall, a comparison of the reaction-coupled flexibility in the two DHFR enzyme folds indicates that motion induces changes in the chemical environment in the active-site, particularly at the C_D_ and C_A_ to facilitate the hydride transfer.

### Nuclease

RNaseA is secreted by the pancreas and catalyzes the hydrolysis of single-stranded RNA. The characteristic shape of RNaseA is formed by a β-sheet in the core, surrounded by several flexible loop regions and α-helices. The active-site is located at the bottom of the inverted β-sheet. A distinctive feature of this fold, different from that of CypA and DHFR, is the linkage of the flexible surface loops through disulphide bonds. The RNaseA fold was selected for investigations as NMR experiments by Loria and coworkers have suggested a link between flexibility and function [10],[64]. Moreover, the rate of enzyme conformational changes (∼1,000–3,000 s^−1^) coincides with the enzyme turnover rate (2–3,000 s^−1^) [59].

**Figure 5:**
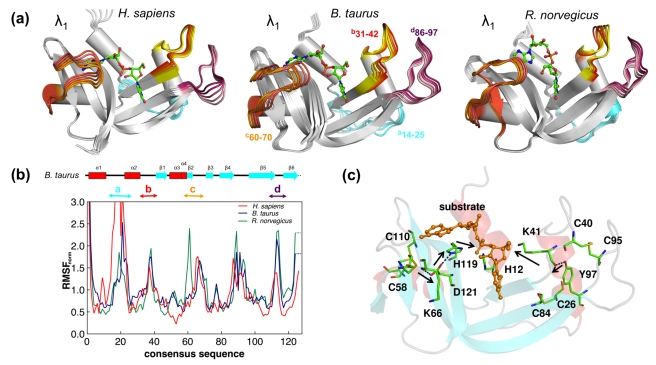
Conservation of reaction-coupled flexibility in enzyme RNaseA across three species. (a) Slowest mode coupled to RNA hydrolysis shows large fluctuations in the same regions (near and away from the active-site) of the enzyme from three species. (b) Enzyme backbone flexibility depicted as normalized RMSF. (c) Conservation of the network interactions (black arrows) connecting the flexible regions as a part of the RNaseA fold (only *B. taurus* RNaseA is shown). The modes are depicted/colored and the RMSF is normalized similarly to the CypA results.

Quantitative estimates of the top 10 reaction-coupled modes (see Table 5) for the three species investigated (*H. sapiens*, *B. taurus*, and *Rattus norvegicu*s) show 67%–70% similarity. Further, the slowest modes also show remarkably similar displacements in surface loop areas near the active-site as well as distal to the active-site (see Movies S7–S9). Note that these modes correspond to the slow conformational fluctuations of the entire enzyme–substrate complex. The dynamical clustering shows the presence of three clusters (the β-sheet forming two clusters and the loops and a helix forming an additional cluster; see Figure S8). Dynamical cross-correlations between residues and enzyme flexibility are also preserved over evolution (see Figure 5, Figures S9–S10).

**Table 5:** Similarity of the slowest modes in RNaseA.

Species	*B. taurus*	*R. norvegicus*
*H. sapiens*	0.677	0.730
*B. Taurus*		0.666

The subspace overlap was based on the Hess' metric for the top 10 modes in the reactant-product ensemble.

A network of interactions coupled to catalysis and connecting the regions of high flexibility also appears to be present in RNaseA. Similar to the CypA and DHFR network, this network is formed by the connection of the surface loop regions all the way to the active-site. In RNaseA, the highly flexible surface regions are linked to other loops through disulphide linkages (C26–C84, C40–C95, C58–C110 in *B. taurus*) and a hydrogen bond (Y97O_η_–K41O). Conserved residues in the active-site (H12 and H119) mediate these network motions between the enzyme and the substrate. These linkages are conserved over evolution as a part of the enzyme fold.

#### Impact of missing network on enzyme catalysis

**Figure 6:**
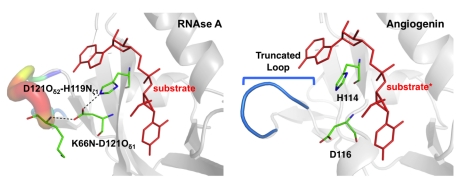
The missing RNaseA network in human angiogenin. Comparison of RNaseA (from *B. taurus*) and human angiogenin (based on PDB structure 2ANG and a modeled substrate based on 1U1B, indicated by *) shows similar fold with similar active-site residues; however, angiogenin shows a truncated network loop. This flexible surface loop in RNaseA forms a hydrogen bond with dynamically important network residue D121.

The human angiogenin protein provides an interesting comparison with the chemistry catalyzed by RNaseA. This protein also catalyzes the hydrolysis of single-stranded RNA but with lower catalytic efficiency than RNaseA (at rates 10^4^–10^6^ less) [65]. Angiogenin is structurally similar to RNaseA (76% similarity in sequence); the active-site shows similar contacts with the substrate, including residues H13, K40, and H114 located in positions structurally equivalent to the catalytically important H12, K41, and H119 in RNaseA [66]. However, its major difference with RNaseA is the truncation of a surface loop located >10 Å from the active-site region (see Figure 6). This surface loop forms an important part of the network in RNaseA; residue K66 on this loop interacts with another network residue, D121. Located in the vicinity of the active-site, the dynamical motions of D121 have been implicated in catalysis, and mutation of this residue results in 90% activity loss in RNaseA [67].

## Discussion

An integrated view of protein structure, flexibility, and function is emerging to support a better understanding of the detailed biophysical mechanism of enzyme catalysis [15]. The role of conserved structural interactions between active-site residues and substrate has been understood for some time [8]; however, the role of the overall enzyme fold remains a mystery, particularly the conserved residues that are located far from the active-site. Increasing evidence continues to link protein motions with designated functions, including enzyme catalysis. The intrinsic flexibility of a protein is related to the overall shape (fold), as well as the local organization of dynamical regions. Does all the emerging evidence suggest that the overall enzyme fold is optimized for structural as well as dynamical effects to carry out the protein function?

**Figure 7:**
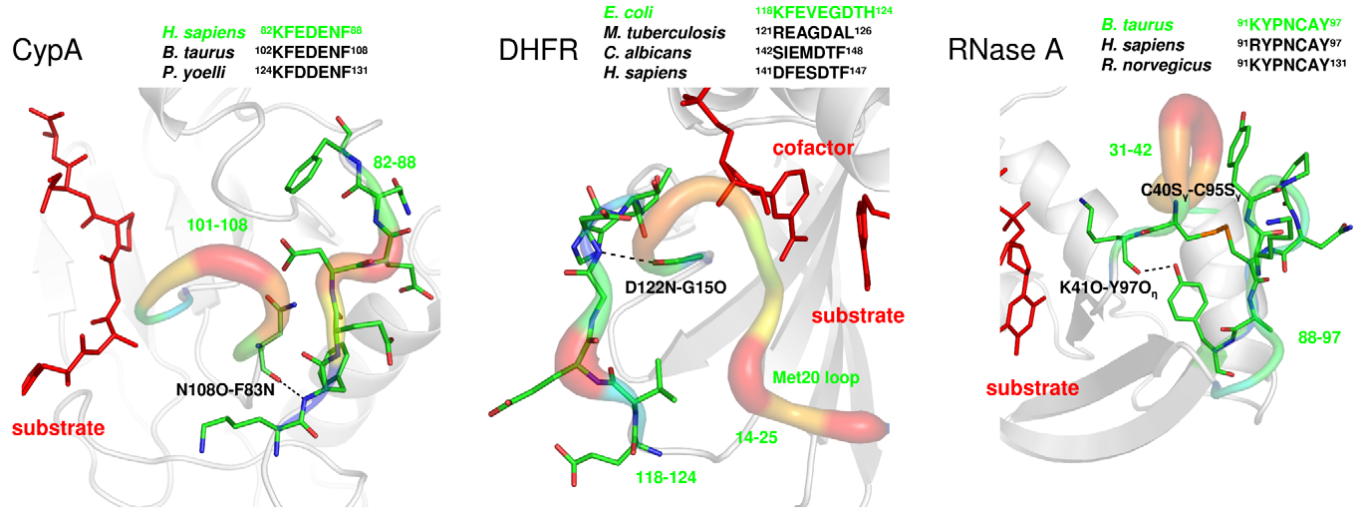
Common features of the network of promoting motions/vibrations across enzyme folds. The three enzyme folds investigated show the presence of highly flexible surface loop regions, which are connected to another flexible region in close proximity to the active-site (flexible regions are shown as tubes and colored to indicate degree of flexibility). These surface loops show high flexibility in X-ray structure (temperature factors) and show the presence of residues with long side-chains and are interconnected to the active-site through preserved hydrogen bonds.

Careful characterization of the networks discovered in the CypA, DHFR, and RNaseA enzyme fold displays common features (see Figure 7). The networks discovered connect surface loop regions to conserved active-site residues that make direct contacts with the substrates. The surface loop regions show a high degree of flexibility as observed in X-ray and NMR investigations as well as computational studies. These regions are exposed to the solvent and contain non-conserved residues with long side-chains, possibly to increase the solvent–enzyme thermodynamical coupling. Another common feature observed in these networks is the connection of these flexible loops, through a conserved hydrogen bond, with another region at the edge of the active-site. It has been reported that bulk solvent fluctuations drive internal protein dynamics, thus impacting protein function [37]. In other words, the discovered networks could serve as a mechanism for coupling of the hydration-shell solvent to the chemical step [26]. Previous investigations have also suggested the existence of conserved energy pathways as a part of protein structure [68]. NMR studies of RNaseA have also revealed that a distant loop (residues 14–25) modulates the active-site motions [10]. Recent computational studies also provide insights into the hierarchy of internal motions, spanning the entire structure, which enable enzyme to visit conformational sub-states [69]. Further, these studies also indicate that some of these conformational sub-states contain geometrical features for the progress of the reaction mechanism.

### Linking Protein Flexibility to Enzyme Catalysis

The detailed biophysical mechanism of how protein motions influence enzyme catalysis remains a topic of intense debate. The wide range of time-scales of protein motions and the narrow (but improving) resolution windows of experimental techniques have presented challenges [2]–[3],[21]. Atomic-level information at different time-scales provided by the computational methods, as highlighted here, could be useful in guiding experimental investigations. The conservation of distally located dynamical regions in enzymes could be argued as coincidental; however, mutations in these regions (located far away from the active-sites) can have a significant impact on the enzyme mechanism and/or kinetics. In *E. coli* DHFR, mutation of network residues (D122 and nearby G121 located >10 Å from the active-site) has shown alteration in enzyme kinetics [30],[58]. Modulation of activation energy barrier has been observed based on computational studies of slow conformational fluctuations [70]. Single-molecule experiments of wild type and G121 and/or M42 mutants have also provided additional evidence [28]. In R67 DHFR, the enzyme kinetics can be positively or negatively impacted based on single or double mutation of K32 (located >13 Å from the active-site) corresponding to different sub-units [62],[71]. In CypA, fascinating details of the conformational changes in the network residues associated with the catalytic step have been obtained including details of minor conformation populations [21],[36]. For Pin1, a number of residues in loop 63–82 (located >10 Å from the active-site) have been observed to be essential for function [55]. In RNaseA, the millisecond dynamics of network residue D121 has been linked to catalysis [64],[67]. As an interesting protein design application, the discovery of network in DHFR has also led to designing of a channel of allosteric communications in protein complexes [72]. All the above-mentioned regions are dynamically important parts of the conserved networks described in this article. Note, investigations have confirmed that the observed impact of distal mutations is not due to changes in the enzyme structure but due to changes in protein motions [21],[36],[73]–[74]. Collectively, these previous observations and our findings possibly explain why there can be a drastic impact on enzyme activity when these crucial network interactions, even when located far from the active-site, are altered.

Quantitative estimates of the impact of protein motions on enzyme mechanism have been difficult to obtain. Computational investigations of hydride transfer catalyzed by *E. coli* DHFR indicate 1–3 kcal/mol contributions of individual collective motions on the activation energy barrier, which is ≈13.4 kcal/mol [75]–[76]. Further, enzyme motions also indicate impact on the transition state barrier recrossings in *E. coli* DHFR (transmission coefficient calculated to be 0.80) [29]. A frequently raised concern is that the observed motions could be caused by catalysis. With the information available at present, it is difficult to completely isolate the cause and effect; however, NMR and X-ray studies indicate that some of the enzyme motions are present intrinsically, even in the absence of substrate or non-productive complexes [20],[62].

The emerging evidence leads us to ask the question, what features of the enzyme fold are optimized for the targeted reaction? The evidence presented in this study suggests that in addition to an enzyme fold serving as a scaffold to orient the active-site residues, the conformational flexibility of distal regions also impacts the protein function. Similar to the conservation of structurally important residues, the movements of important regions are also conserved across species ranging from single-cell organisms to complex life forms. Enzyme structures with similar sequences are expected to show similar intrinsic flexibility due to similar molecular architecture [45]–[48]. Therefore, it could potentially be argued that the conservation of flexibility across different species could purely be a coincidence due to the similarity in shape. Note that the conserved flexibility discussed in this article focuses not on the global conformational fluctuations associated with the overall structure but *specifically on the slow conformational motions coupled to the rate-limiting enzyme catalysis step* (which are not necessarily the same as intrinsic slowest movements of the enzyme folds). Moreover, the reaction-coupled slow conformational fluctuations are conserved across multiple species for three entirely different folds catalyzing diverse types of chemical reactions. Note, the results reported in this study are consistent with previous discoveries of network of promoting motions [12] and that reaction-promoting motions are intrinsic properties of enzymes [36].

Enzymes that catalyze the same chemistry but have no sequence or structural similarity also show a remarkably similar impact of distal motions on the enzyme mechanisms. This indicates that the conservation of flexibility may be designed into the molecular architecture of enzymes, similar to conservation of structural elements. Therefore, the results presented here provide a counterpoint that the reaction-coupled flexibility may not be a coincidence but poses an additional constraint on conservation of the enzyme fold. For homologous enzymes, the conservation of enzyme fold (or the overall shape) may possibly be also due to the conservation of flexibility of distal areas linked to the enzyme mechanism. Even if it may appear obvious that homologous enzyme folds have similar dynamics, the findings reported here may explain why modifications to the overall molecular shape may not be tolerated (for example, the truncation of a distal surface loop in human angiogenin). Therefore, the identification of distal areas with reaction-coupled flexibility has implications for allostery and protein engineering.

To summarize, the interconnection between enzyme fold, flexibility, and function presented here suggests that the conventional emphasis that *structure encodes function* may need to be expanded to better understand the fundamental mechanisms of how enzymes work. Conservation of reaction-coupled conformational flexibility as an important characteristic of the enzyme fold suggests that *structure encodes dynamics* and together *structure–dynamics encode function*. It is entirely possible that specific enzymes have evolved to utilize the structural interactions with flexibility making only minor contributions [4],[7]. In other systems such as CypA, DHFR, and RNaseA, the contributions of flexibility could be closely related to the enzyme mechanism. This emerging view of proteins provides some basis for understanding allosteric and cooperative effects and has wide implications for drug design, as well as protein engineering.

## Material and Methods

Enzyme–substrate complexes were modeled using molecular mechanics under explicit solvent conditions, as previously described [25]. The AMBER simulation package was used for model building and simulations [77]. AMBER's *parm98* force-field and SPC/E water model were used. Note, in the previous work, we have verified the suitability of the *parm98* force-field for enzyme dynamics modeling in comparison with other popular force-fields [25]. The starting structures for the enzymes were obtained from the protein data bank. Table 6 summarizes the sequence identity and the structural similarity for the enzyme structures used in this study. After the model preparation of enzyme–substrate in explicit water, the system was equilibrated based on the protocol described previously. To briefly summarize, the model was minimized to remove bad contacts and slowly heated to 300 K. All production runs were performed at 300 K under constant volume and energy (NVE) conditions.

**Table 6:** Sequence and structural comparison of the enzymes investigated.

CypA	CypA (1IHG)	CypA (1Z81)		DHFR	DHFR (1DG5)	DHFR (1AI9)	DHFR (1KMV)	RNaseA	RNaseA (2K11)	RNaseA (1RRA)	
**1AWQ**	63^a^		54	**1RX2**	36	31	30	**1U1B**	69	67	
	29.5^b^		28.1		23.6	20.2	21.1		18.5	22.2	
	1.1^c^		1.1		1.5	2.2	1.8		1.9	1.1	
**1IHG**			58	**1DG5**		28	31	**2K11**		65	
			29.1			18.9	21.2			18.3	
			1.4			2.6	1.7			2.2	
				**1AI9**			35				
							22.1				
							2.3				

Alignments performed with DaliLite pair wise comparison web tool: http://www.ebi.ac.uk/DaliLite/. See text for the PDB accession codes. ^a^ sequence identity(%), ^b^ Z-score, ^c^ RMSD in the reference PDB structures (Å).

### PPIase

The human cyclophilin was modeled as previously described with the peptide substrate *His–Ala–Gly–Pro–Ile–Ala* [25]. For the *B. taurus* cyclophilin 40 (PDB code: 1IHG), only the residues 2–185 (corresponding to the CypA fold) in the PDB file were used for the model; a substrate peptide *Ala–Gly–Pro–Phe* was modeled on alignment of active-site residues from human CypA. For *P. yoelii* cyclophilin (PDB code: 1Z81), only the residues 40–210 in the PDB file were used for the model; a substrate peptide *His–Val–Gly–Pro–Ile–Ala* was modeled on alignment of active-site residues from human CypA. The reaction pathway was modeled with the amide bond dihedral angle (ω) as reaction coordinate; 37 windows (in 5° decrements) were used to map the reaction from the reactant state (ω  = 180°) to the product state (ω  = 0°). Each window was simulated for 200 ps, and 500 structures from each molecular dynamics (MD) simulation were collected for the identification of the reaction-coupled flexibility and correlated motion analysis. Therefore, a total of 18,500 conformations were used for clustering analysis, motion analysis, and computations of the reaction-coupled modes. A similar protocol was used for the modeling of Pin1. Pin1 was modeled based on human Pin1 X-ray crystal structure (PDB code: 1PIN). Only the PPIase domain, residues 45–163, was used for model building. Model substrate pSer–Pro was modeled based on the position of the peptide Ala–Pro present in the X-ray structure. Note that the free energy profiles for the *cis/trans* isomerization catalyzed by CypA were previously characterized by our group and published elsewhere [25],[78].

### Oxidoreductase

The *E. coli* DHFR (PDB code: 1RX2) was modeled as described previously [29]. The cofactor NADPH present in the PDB file was included in the model, and the substrate DHF was modeled based on the folate molecule present in the PDB file. For *M. tuberculosis*, *C. albicans*, and *H. sapiens* DHFR, the models were prepared based on the PDB coordinates (PDB codes: 1DG5, 1AI9 (chain A only) and 1KMV, respectively), the cofactor was taken from PDB files, and the substrate was modeled based on *E. coli* DHFR. For modeling the hydride transfer step, we used protonated substrate and the empirical valence bond (EVB) method, which was developed by Warshel and coworkers [79]–[80]. The modeled enzyme reaction is the hydride transfer from NADPH (cofactor) to protonated DHF to produce NADP^+^ and THF. The present study involves the modeling of the hydride transfer from the C4N carbon on the cofactor (C_D_) to the C6 carbon on the protonated substrate DHF (C_A_). The EVB method, in combination with classical molecular mechanics, was used for sampling of the conformations along the hydride transfer reaction. A total of 21,000 conformations were collected representing the enzyme–substrate conformations sampled along the reaction pathway. These conformations were used for clustering analysis, motion analysis, and computations of the reaction-coupled modes. A similar protocol was used for R67 DHFR. The protocol used for simulation of hydride transfer using EVB method was the same as described in [62].

### Nuclease

For *B. taurus* RNaseA, using the coordinates from PDB (PDB code: 1U1B chain A) and substrate RNA with sequence UA was modeled based on the ligand molecule present in the PDB file as well in another related PDB structure (1RCN). The product state was modeled with the hydrolyzed bond based on the above procedure. For *R. norvegicus* RNaseA and *H. sapiens* pancreatic ribonuclease, we used coordinates from PDB (PDB codes: 1RRA and 2K11 (NMR model 10), respectively). The substrate UA was modeled based on *B. taurus* substrate coordinates. After equilibration, 5 ns of MD runs were performed for only the reactant and the product states; 10,000 structures from each state were used for structural and dynamical motion analysis. The 20,000 conformations were used for clustering analysis, motion analysis, and computations of the reaction-coupled modes.

### Reaction-Coupled Flexibility

Protein flexibility at long time-scales or the slow conformational fluctuations in enzyme-substrate complexes were identified using quasi-harmonic analysis (QHA) [25],[81]. QHA captures the large-scale conformational fluctuations within a collection of conformations by diagonalizing the mass-weighted covariance matrix known as the atomic fluctuation matrix (*F_αβ_*). For a system with N atoms, *F_αβ_* is a 3N × 3N symmetric matrix, defined as shown below: 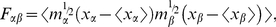
(1)where α and β represent the 3N degrees of freedom in Cartesian space; *m_α_* is the mass of the atom and the quantity within 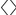 denotes an average over the ensemble of structures in MD simulation. The inverse square root of the eigenvalues determined by diagonalizing *F_αβ_* represent the frequencies associated with protein eigenmodes (λ). The eigenvectors represent the displacement vectors of the individual atoms. The lowest frequencies correspond to large-scale cooperative motions in the protein; the higher frequencies represent localized motions.

For the enzyme–substrate complexes used in this study, ***F*** is constructed from system snapshots traversing the entire reaction pathway (or combination of the reactant and product state in case of RNaseA). To focus on the enzyme–substrate motions, the water molecules were excluded from the QHA calculations. QHA of the entire set of enzyme-complex conformations sampled along the reaction pathway allows identification of conformational fluctuations occurring at the time-scale of the reaction. The slowest modes of QHA (λ_N_, ranked by eigenvalues) correspond to the conformational fluctuations observed in protein at long time-scales. Note that Figures 1, 3, and 5 provide a list of λ_N_ corresponding to the modes showing the largest coupling to the catalyzed reaction. Protein regions showing similar motions over the course of the reaction pathway were identified using a clustering methodology [82]. See Figure S11 and Text S1 for details of methodology for dynamical clustering and cross-correlations.

For analysis of motions and conformational fluctuations 18,500 CypA conformations and 21,000 DHFR conformations collected along the entire reaction profile were used. The slowest 50 (top 50 eigenmodes based on smallest eigenvalues) QHA modes were analyzed for coupling with the reaction; as previous studies indicated, the slowest 10 QHA modes can capture most of the protein motions at microsecond time-scales (∼78%) [83]. Note that the analysis of the larger sub-set of the eigenmodes indicated that modes with higher eigenvalues show motions that are localized in specific regions of the protein. Only the eigenmodes corresponding to global conformational changes with the largest coupling to the reaction pathway were characterized in detail. For RNaseA only the reactant and product states were used, with a total of 20,000 conformations, and only the slowest modes were analyzed. The use of end-states only (as in the case of RNaseA) provides a qualitative estimation of the reaction-coupled flexibility, as discussed in a recent study [83].

A quantitative measure of similarity of reaction-coupled flexibility for homologous enzymes across different species was obtained by computing the sub-space overlap for top 10 reaction-coupled modes, based on the Hess' metric [52], defined as:
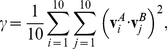
(2)where γ is the overlap in the sub-space spanned by the eigenvectors 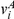 and 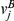 coupled to the reaction. The super-scripts on the eigenvectors represent the individual species A and B, respectively. Note only the top 10 eigenvectors that are coupled to the reaction were used in this computation. The values of γ can range between 0 and 1, with the values close to 1 indicating largely similar sub-spaces (indicating similarity in motions), whereas the values close to zero indicate that there is no similarity in the motions.

### Definition of Modes Coupled to the Reaction

**Figure 8:**
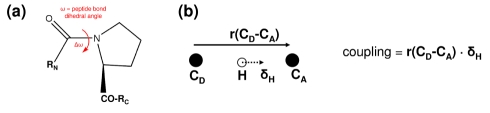
Definition of reaction coupling. (a) Coupling to the *cis/trans* isomerization catalyzed by CypA, with ω indicating the amide bond dihedral angle. For mode *i*, the coupling Δω is defined as: Δω_i_  =  ω_i_^+^ − ω_i_^−^, where ω^+^ and ω^−^ are the two extreme ends of the displacement, computed by projecting the eigenmodes on the entire conformational ensemble. (b) Coupling to hydride transfer catalyzed by DHFR. r(C_D_–C_A_) represents the position vector to acceptor carbon from donor carbon, and δ_H_ is the displacement vector associated with hydride in the eigenmode.

See Figure 8 for the definitions. In CypA and Pin1, the degree of coupling with the reaction was defined as the variation in amide bond dihedral angle (ω) in the mode. For each eigenmode computed from QHA, the variation in the dihedral angle (Δω) was computed as a measure of degree of coupling to the reaction coordinate. In DHFRs, the coupling was defined as the dot product of the hydride transfer displacement vector in the eigenmodes with the C_D_–C_A_ distance vector. Note that QHA provides the displacement vectors associated with atoms (for each eigenmode); the amplitude of displacement is determined by the range observed in the entire conformational ensemble through projection of the modes on the conformational snapshots. The analyzed modes correspond to the time-scale of reaction (∼0.1 ms in CypA and ∼1 ms in DHFR).

### Network and Dynamic Correlations

The networks of protein vibrations/motions were identified by characterization of enzyme regions displaying large movements in the QHA modes, by investigating clustering of regions based on similarity in motions as well as dynamic cross-correlation maps, and by monitoring the distances of correlated regions over the course of a reaction, as reported previously [25]. In particular, the large dynamical cross-correlation between different residue pairs followed by structural analysis was used to identify the chain of interactions in the networks. Additionally, as described in Text S1, a new methodology for dynamic clustering was used to identify enzyme regions that exhibit similar dynamical characteristics over the enzyme pathway [82]. Genomic analysis was performed using Clustal-W [84], and the structural analysis was aided by the PyMOL program [85].

### Validation of the Models Used in This Study

The models used in this study for investigating the slow conformational fluctuations in enzymes have been carefully validated. The identification of the reaction-coupled modes is based on the use of QHA of the enzyme–substrate conformations collected over the reaction pathway. As the identification of the slow conformational fluctuations is an important part of our investigations, we have verified the ability of QHA to reproduce experimentally observed protein conformational fluctuations at long time-scales. In previous work, we have verified the ability of QHA analysis to identify the experimentally observed correlated protein motions at microsecond-millisecond time-scales [83]. The slow conformational fluctuations obtained using QHA show >75% similarity with deviations observed in an ensemble of NMR conformations. Figure S12 provides a comparison of computationally obtained enzyme flexibility with the temperature factors (β-factors) from X-ray crystal structures of CypA, DHFR, and RNaseA. The computationally obtained enzyme flexibility (root mean square fluctuations) reproduces the experimentally observed temperature factors. Further, significant agreement between the correlation matrices for enzymes from different species is observed (Figures S2, S6, and S9). This observation indicates convergence of the computational simulations and is consistent with previously reported observations [86]–[87].

Further, previous work on CypA also indicated that the reaction-coupled conformation fluctuations are reproduced in human CypA with three different peptide substrates and a biologically relevant protein substrate [25],[78]. Moreover, the network regions showing large flexibility identified by using our models have also been validated by NMR investigations [36]. Similarly, for DHFR the regions of high conformational flexibility (including Met 20 loop and βF-βG loops) have also been proposed as a part of coupled network of protein motions and validated using NMR and enzyme mutation/kinetics experiments [35],[58],[88]. For RNaseA, the regions of high flexibility also coincide with the observations made from NMR investigations [10].

## Supporting Information

Figure S1 Dynamical clusters in enzyme CypA. Six clusters were identified (shown in different colors), which were observed to be identical across the three species investigated. The red cluster consisting of the substrate and the β-hairpin formed by residues 13–16 (*H. sapiens* sequence number) exhibit large-scale fluctuations. The hydrophobic core of the protein (dark blue) and the active site regions (cyan) and the flexible surface loops along the outer edge of the active site (orange) are similarly clustered across all the three species. The flexible loops behind (yellow) and adjacent (green) to the active site region exhibit coupled motions that are also conserved features of this enzyme fold. Note, regions that are insertions in the other two species (*B. taurus* and *P. yeolii*) are shown in dark gray color. Regions of similar dynamical fluctuations are conserved, indicating that dynamics coupled to the catalytic mechanism are conserved across multiple species regardless of sequence homology.
(TIF)

Figure S2 Cross-correlations observed along the reaction profile for CypA. B1–B8 correspond to the correlations along the β-sheet of the enzyme. H1–H3 correspond to the three α-helices. Regions marked I1–I4 correspond to distal correlations observed along loop structures. I1: residues 29–33 with 85–86, I2: 34–36 with 77–78, I3: 56–57 with 142–150 and I4: residues 82–85 with 104–108. Note, residue numbers mentioned above refer to *H. sapiens* as the reference species; corresponding residue numbers for the two species are available in Table S1.
(TIF)

Figure S3 Conservation of the network interactions as a part of PPIase fold: human CypA (I) PDB code: 1RMH; human cyclophilin B (II) PDB code: 1CYN; *B. Malayi* (III) PDB code: 1A33; *B. Taurus* (IV) PDB code: 1IHG; *E. coli* (V) PDB code: 2NUL. The equivalent hydrogen bonds are listed in Table S2. Substrate is shown in orange ball-and-stick model for human CypA.
(TIF)

Figure S4 Reaction-coupled flexibility in Pin1 PPIase. (a) Top 2 reaction-coupled modes are shown. The regions indicating high flexibility are colored and marked and the substrate pSer–Pro is shown as red sticks. (b) The RMSF for top 10 reaction-coupled modes are shown; the regions corresponding to large displacements in (a) are marked. (c) The dynamical cross-correlation plot computed based on conformations collected over the reaction pathway. There are several regions showing large correlation; however, only the correlations between five regions of enzyme showing large flexibility during reaction are marked. (d) Network of promoting motions coupled to the PPIase reaction in Pin1. Note that only the PPIase domain (residues 45–163) is shown.
(TIF)

Figure S5 Dynamical clusters of residues in enzyme DHFR. Five dynamical clusters were identified across the four species investigated, indicating an identical behavior of flexibility coupled to hydride transfer. The Met20, βF–βG, βG–βH, and the substrate binding loops (cluster shown in orange) exhibit large-scale fluctuations. The central β-sheet is split into two clusters (cyan and dark blue), which is consistent with the observation by Sawaya and Kraut regarding the intrinsic twist in the β-sheet [23]. Further, loops shown in yellow are coupled to the substrate-binding region. The flexibility of these clustered regions is a conserved feature of this enzyme fold as it is similar across species. Regions shown in dark gray (in *M. tuberculosis* and *H. sapiens*) are additional inserts not found in the other species.
(TIF)

Figure S6 Cross-correlations in enzyme DHFR along the hydride-transfer. Regions marked S1–S2, H1–H4 represent the correlated dynamics of the secondary structural elements in DHFR. Regions I1–I3 however correspond to distal correlations observed from the reaction profile. I1: residues 15–22 correlated with 116–125 (Met20 and βF–βG loops), I2: 31–36 (α-helix A) correlated with 142–150 (βG–βH). I3: residues 64–72 (βG–βH) negatively correlated with residues 142–150 (βG–βH). Note, we have used the reference structure as *E. coli* (1RX2) for the residue numbers mentioned above. Corresponding regions from other species are shown in Table S3.
(TIF)

Figure S7 Details of the conserved network of coupled motions in enzyme DHFR. The flexible loops on the surface are connected to the active-site through conserved residues, hydrogen bonds, and hydrophobic interactions as listed in Table S4.
(TIF)

Figure S8 Dynamically coupled clusters in RNaseA. Three identical clusters were identified across the species. The three α-helices are clustered into three regions (blue, green, and cyan), indicating that the dynamics of these helices are quite different. The β-sheet is split into two distinct clusters (green and blue) depending on how these regions flank the substrate in the active site. The opposed movements of the β-sheet regions (see movies) and the motions of the flexible loop regions (cyan and blue regions) are a conserved dynamical feature of the RNaseA fold.
(TIF)

Figure S9 Cross-correlations in RNaseA: Regions H1–H3 and S1–S4 correspond to correlations observed from secondary structural elements (α1–α3, β1–β5), respectively. Regions I1–I2 correspond to distal correlations observed. The distal correlations observed from RNaseA are depicted in Table S5.
(TIF)

Figure S10 Details of conserved networks in RNaseA. The flexible loops on the surface are connected to the active-site through conserved residues and disulphide bonds as listed in Table S6.
(TIF)

Figure S11 The dynamic cluster methodology for identification of protein regions exhibiting similar motions over the course of MD simulation(s).
(TIF)

Figure S12 Validation of the enzyme flexibility. Comparison of the computationally obtained flexibility of the enzyme models (root mean square fluctuations) is compared with the temperature factors (β-factors) from the X-ray structures. The results show reproducible trends in high and low areas of enzyme flexibility.
(TIF)

Movie S1 Mode showing the highest coupling to *cis/trans* isomerization reaction. For more information, see Text S1.
(MPG)

Movie S2 Mode showing the second highest coupling to *cis/trans* isomerization reaction. For more information, see Text S1.
(MPG)

Movie S3 Mode showing the third highest coupling to *cis/trans* isomerization reaction. For more information, see Text S1.
(MPG)

Movie S4 Mode showing the highest coupling hydride transfer reaction. For more information, see Text S1.
(MPG)

Movie S5 Mode showing the second highest coupling hydride transfer reaction. For more information, see Text S1.
(MPG)

Movie S6 Mode showing the third highest coupling hydride transfer reaction. For more information, see Text S1.
(MPG)

Movie S7 RNaseA mode with the lowest eigenvalue. For more information, see Text S1.
(MPG)

Movie S8 RNaseA mode with the second lowest eigenvalue. For more information, see Text S1.
(MPG)

Movie S9 RNaseA mode with the third lowest eigenvalue. For more information, see Text S1.
(MPG)

Table S1 CypA regions showing high correlations.
(DOC)

Table S2 Network interactions in PPIase fold.
(DOC)

Table S3 DHFR regions showing high correlations.
(DOC)

Table S4 Network interactions in DHFR fold.
(DOC)

Table S5 RNaseA regions showing high correlations.
(DOC)

Table S6 Network interactions in RNaseA fold.
(DOC)

Text S1 Detailed results of Pin1 computational modeling, details of clustering methodology of protein regions based on conformational fluctuations, and details of the computational methodology used for calculating the dynamical cross-correlation maps are provided.
(DOC)

Text S2 Consensus sequences for the three types of enzymes.
(XLS)

## Abbreviations

CypA: cyclophilin A
DHF: dihydrofolate
DHFR: dihydrofolate reductase
EVB: empirical valence bond
NADPH: nicotinamide adenine dinucleotide phosphate
NMR: nuclear magnetic resonance
p-ABG: para-aminobenzoylglutamate
PPIase: peptidyl-prolyl isomerase
QHA: quasi-harmonic analysis
RNaseA: ribonuclease A
