# Supplementary figures and images for: Evolutionarily Conserved Linkage between Enzyme Fold, Flexibility, and Catalysis
Source: PLoS Biol. 2011 Nov 8;9(11):e1001193. doi: 10.1371/journal.pbio.1001193 (PMC3210774; doi:10.1371/journal.pbio.1001193)

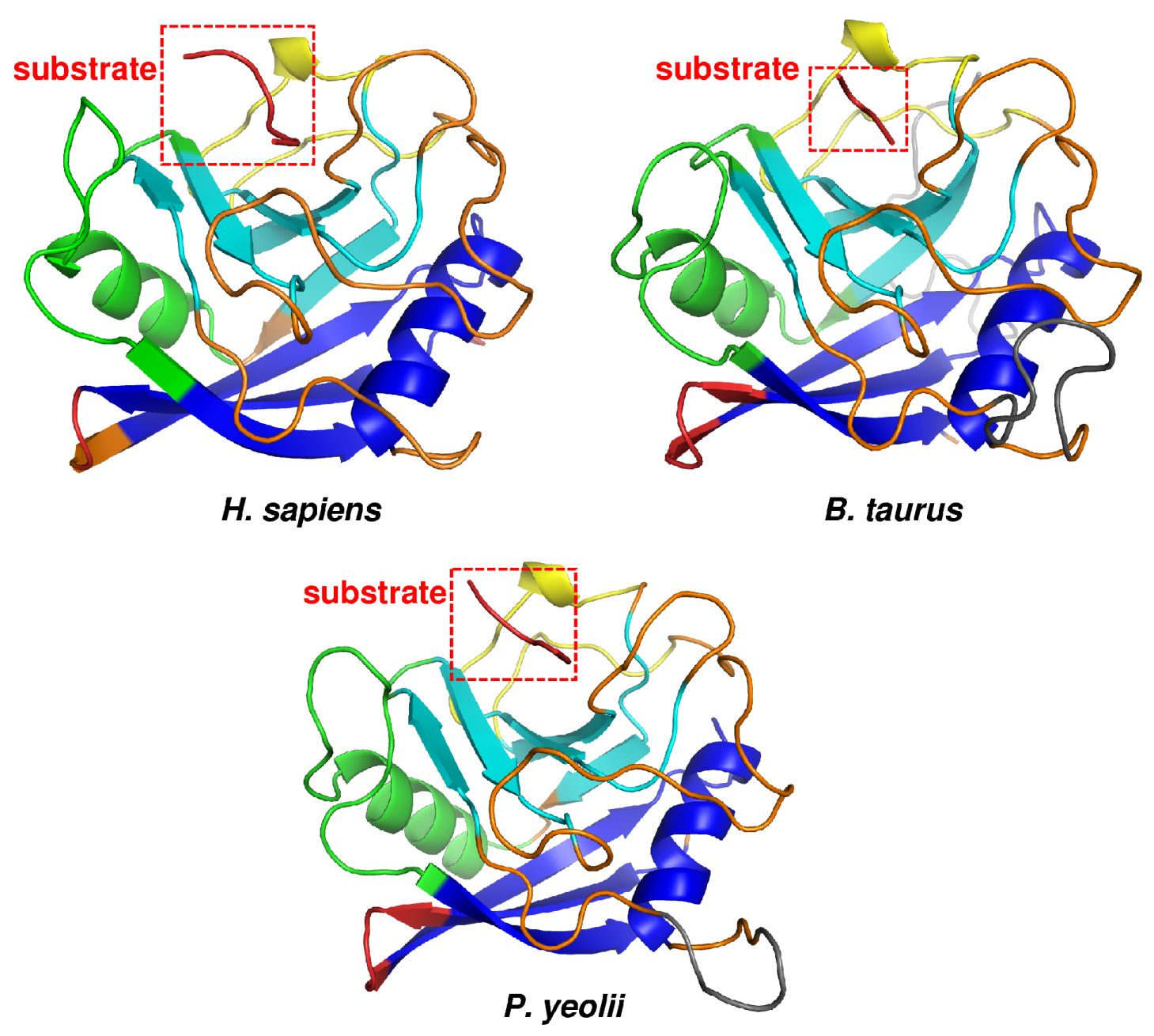

Supplement: Figure S1 — Dynamical clusters in enzyme CypA. Six clusters were identified (shown in different colors), which were observed to be identical across the three species investigated. The red cluster consisting of the substrate and the β-hairpin formed by residues 13–16 (H. sapiens sequence number) exhibit large-scale fluctuations. The hydrophobic core of the protein (dark blue) and the active site regions (cyan) and the flexible surface loops along the outer edge of the active site (orange) are similarly clustered across all the three species. The flexible loops behind (yellow) and adjacent (green) to the active site region exhibit coupled motions that are also conserved features of this enzyme fold. Note, regions that are insertions in the other two species (B. taurus and P. yeolii) are shown in dark gray color. Regions of similar dynamical fluctuations are conserved, indicating that dynamics coupled to the catalytic mechanism are conserved across multiple species regardless of sequence homology. (TIF) [file pbio.1001193.s001.tif]

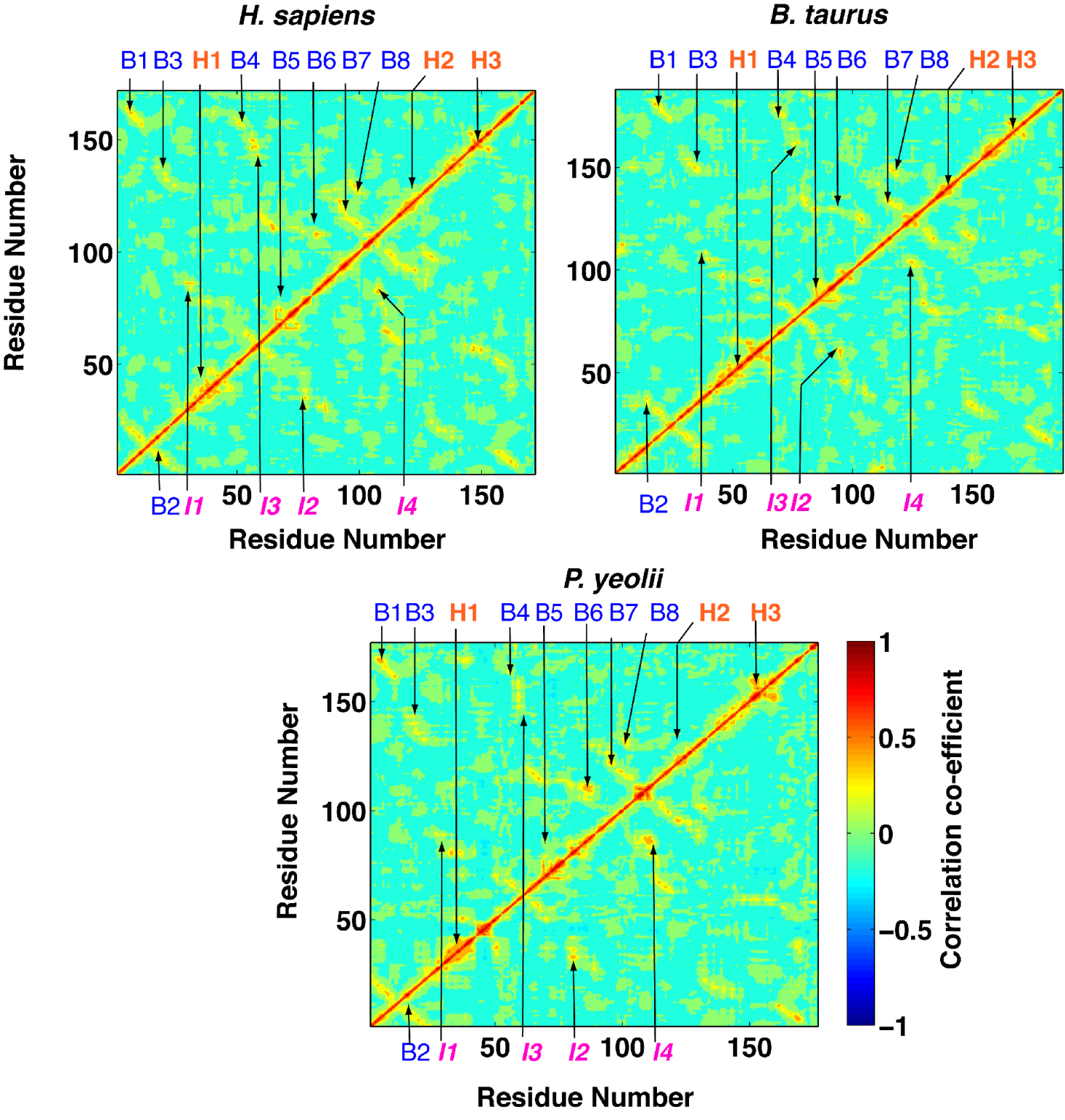

Supplement: Figure S2 — Cross-correlations observed along the reaction profile for CypA. B1–B8 correspond to the correlations along the β-sheet of the enzyme. H1–H3 correspond to the three α-helices. Regions marked I1–I4 correspond to distal correlations observed along loop structures. I1: residues 29–33 with 85–86, I2: 34–36 with 77–78, I3: 56–57 with 142–150 and I4: residues 82–85 with 104–108. Note, residue numbers mentioned above refer to H. sapiens as the reference species; corresponding residue numbers for the two species are available in Table S1. (TIF) [file pbio.1001193.s002.tif]

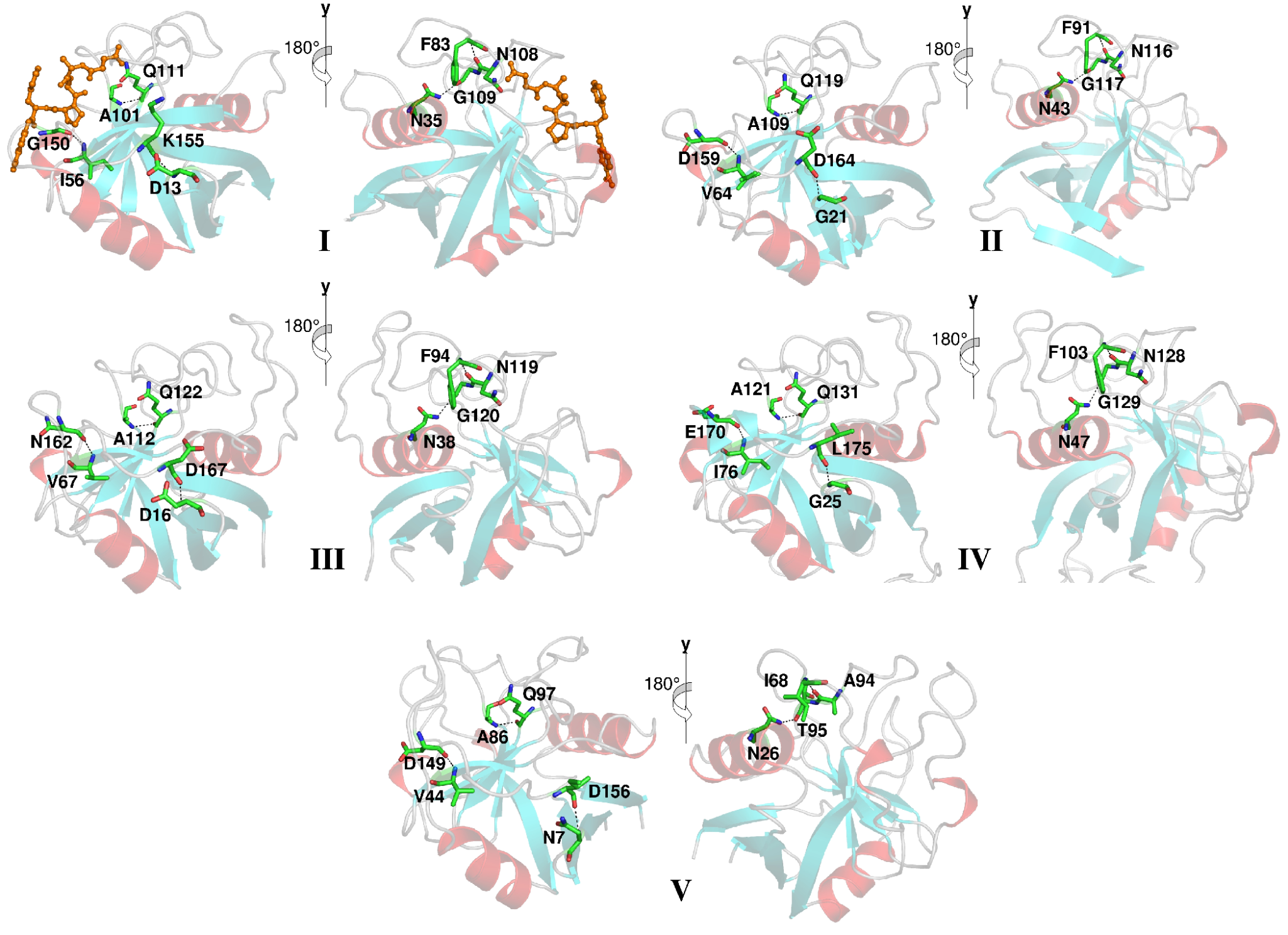

Supplement: Figure S3 — Conservation of the network interactions as a part of PPIase fold: human CypA (I) PDB code: 1RMH; human cyclophilin B (II) PDB code: 1CYN; B. Malayi (III) PDB code: 1A33; B. Taurus (IV) PDB code: 1IHG; E. coli (V) PDB code: 2NUL. The equivalent hydrogen bonds are listed in Table S2. Substrate is shown in orange ball-and-stick model for human CypA. (TIF) [file pbio.1001193.s003.tif]

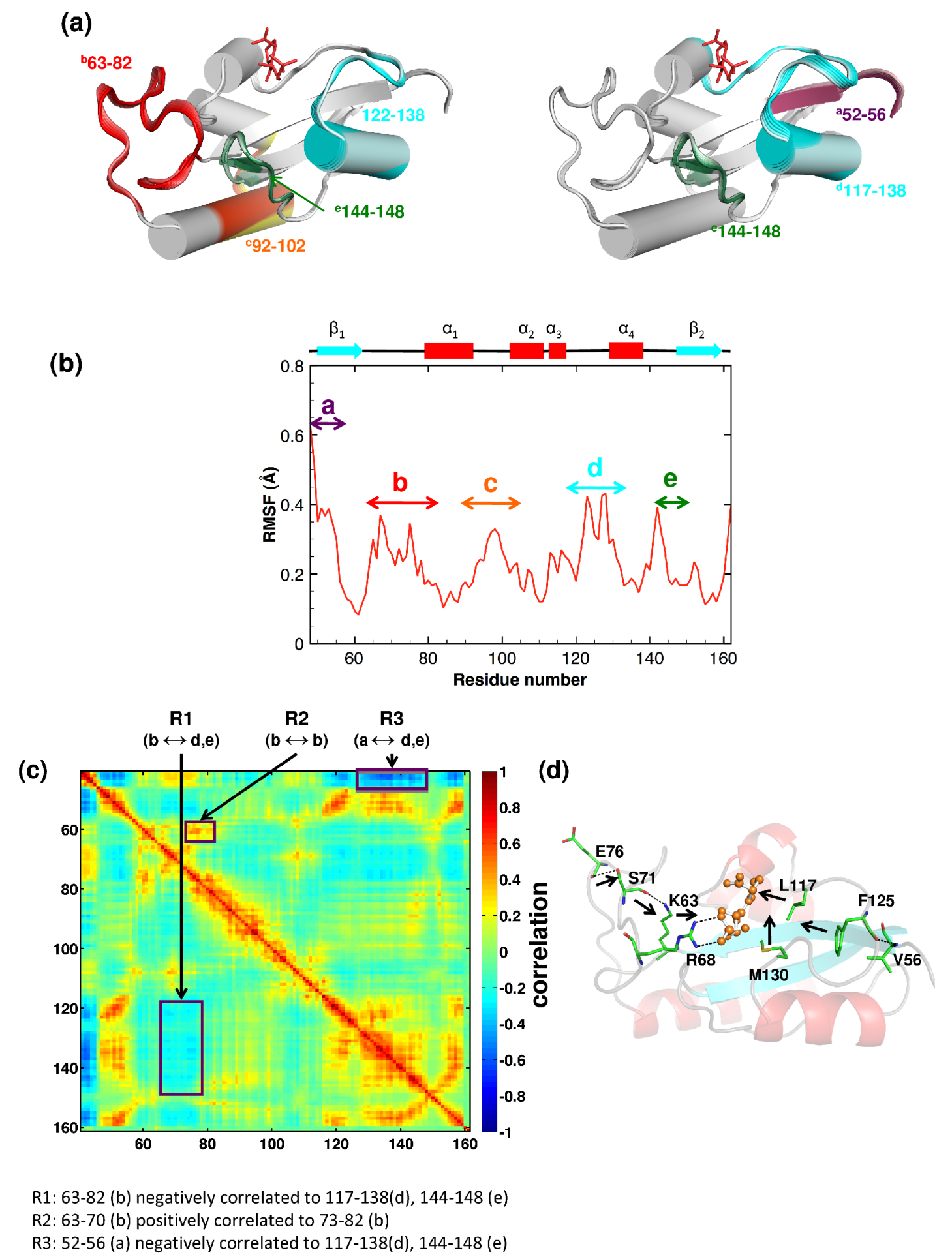

Supplement: Figure S4 — Reaction-coupled flexibility in Pin1 PPIase. (a) Top 2 reaction-coupled modes are shown. The regions indicating high flexibility are colored and marked and the substrate pSer–Pro is shown as red sticks. (b) The RMSF for top 10 reaction-coupled modes are shown; the regions corresponding to large displacements in (a) are marked. (c) The dynamical cross-correlation plot computed based on conformations collected over the reaction pathway. There are several regions showing large correlation; however, only the correlations between five regions of enzyme showing large flexibility during reaction are marked. (d) Network of promoting motions coupled to the PPIase reaction in Pin1. Note that only the PPIase domain (residues 45–163) is shown. (TIF) [file pbio.1001193.s004.tif]

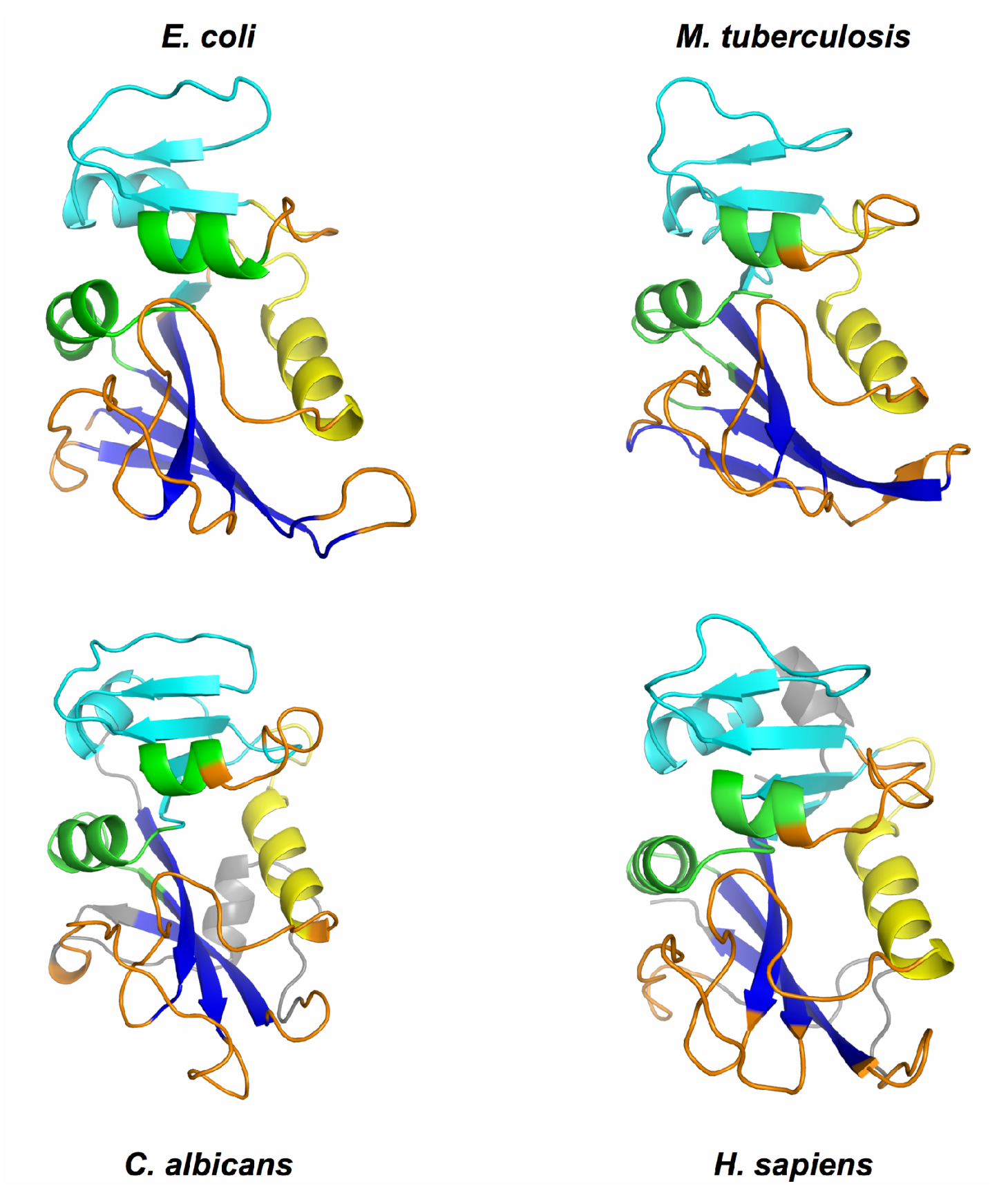

Supplement: Figure S5 — Dynamical clusters of residues in enzyme DHFR. Five dynamical clusters were identified across the four species investigated, indicating an identical behavior of flexibility coupled to hydride transfer. The Met20, βF–βG, βG–βH, and the substrate binding loops (cluster shown in orange) exhibit large-scale fluctuations. The central β-sheet is split into two clusters (cyan and dark blue), which is consistent with the observation by Sawaya and Kraut regarding the intrinsic twist in the β-sheet [23]. Further, loops shown in yellow are coupled to the substrate-binding region. The flexibility of these clustered regions is a conserved feature of this enzyme fold as it is similar across species. Regions shown in dark gray (in M. tuberculosis and H. sapiens) are additional inserts not found in the other species. (TIF) [file pbio.1001193.s005.tif]

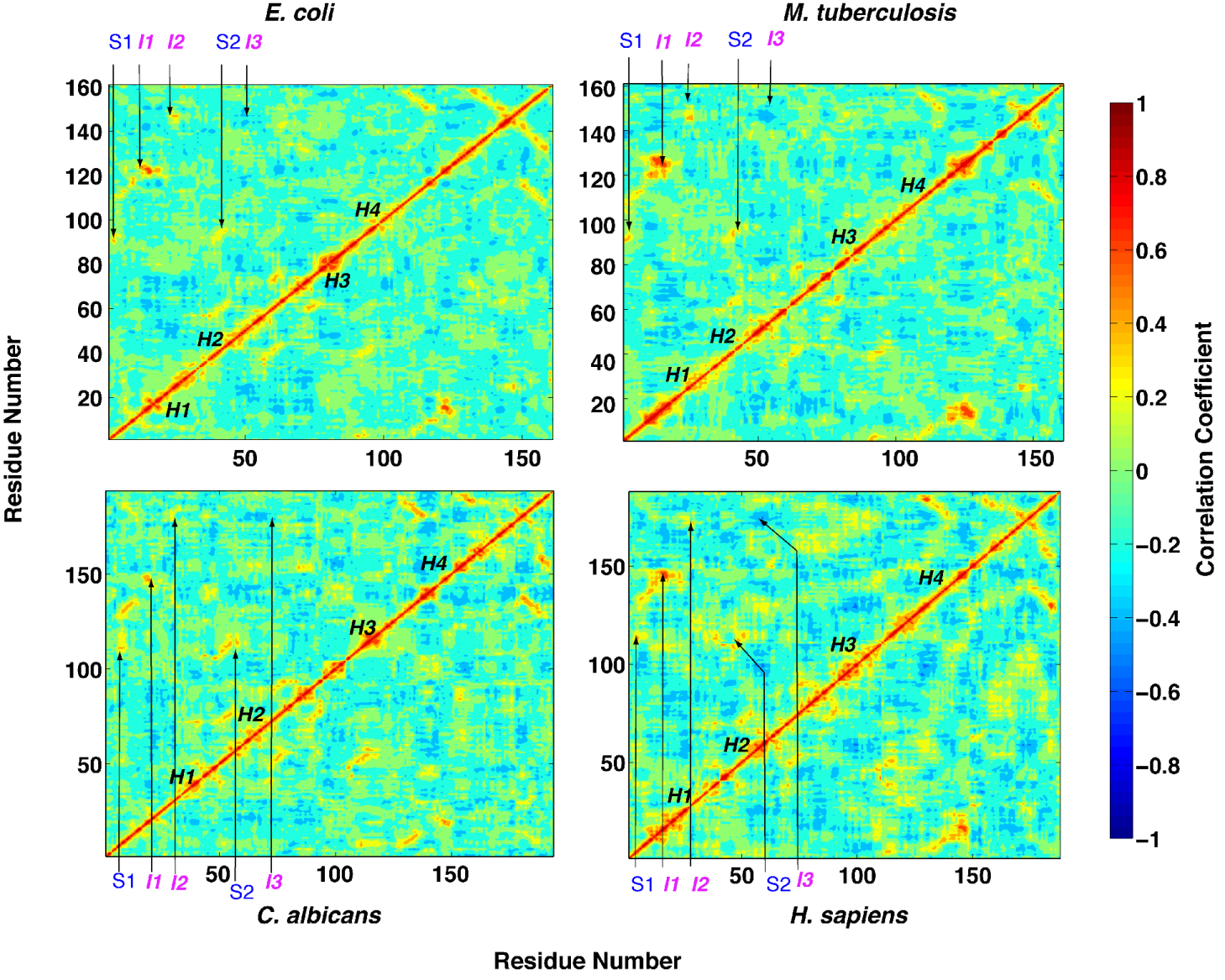

Supplement: Figure S6 — Cross-correlations in enzyme DHFR along the hydride-transfer. Regions marked S1–S2, H1–H4 represent the correlated dynamics of the secondary structural elements in DHFR. Regions I1–I3 however correspond to distal correlations observed from the reaction profile. I1: residues 15–22 correlated with 116–125 (Met20 and βF–βG loops), I2: 31–36 (α-helix A) correlated with 142–150 (βG–βH). I3: residues 64–72 (βG–βH) negatively correlated with residues 142–150 (βG–βH). Note, we have used the reference structure as E. coli (1RX2) for the residue numbers mentioned above. Corresponding regions from other species are shown in Table S3. (TIF) [file pbio.1001193.s006.tif]

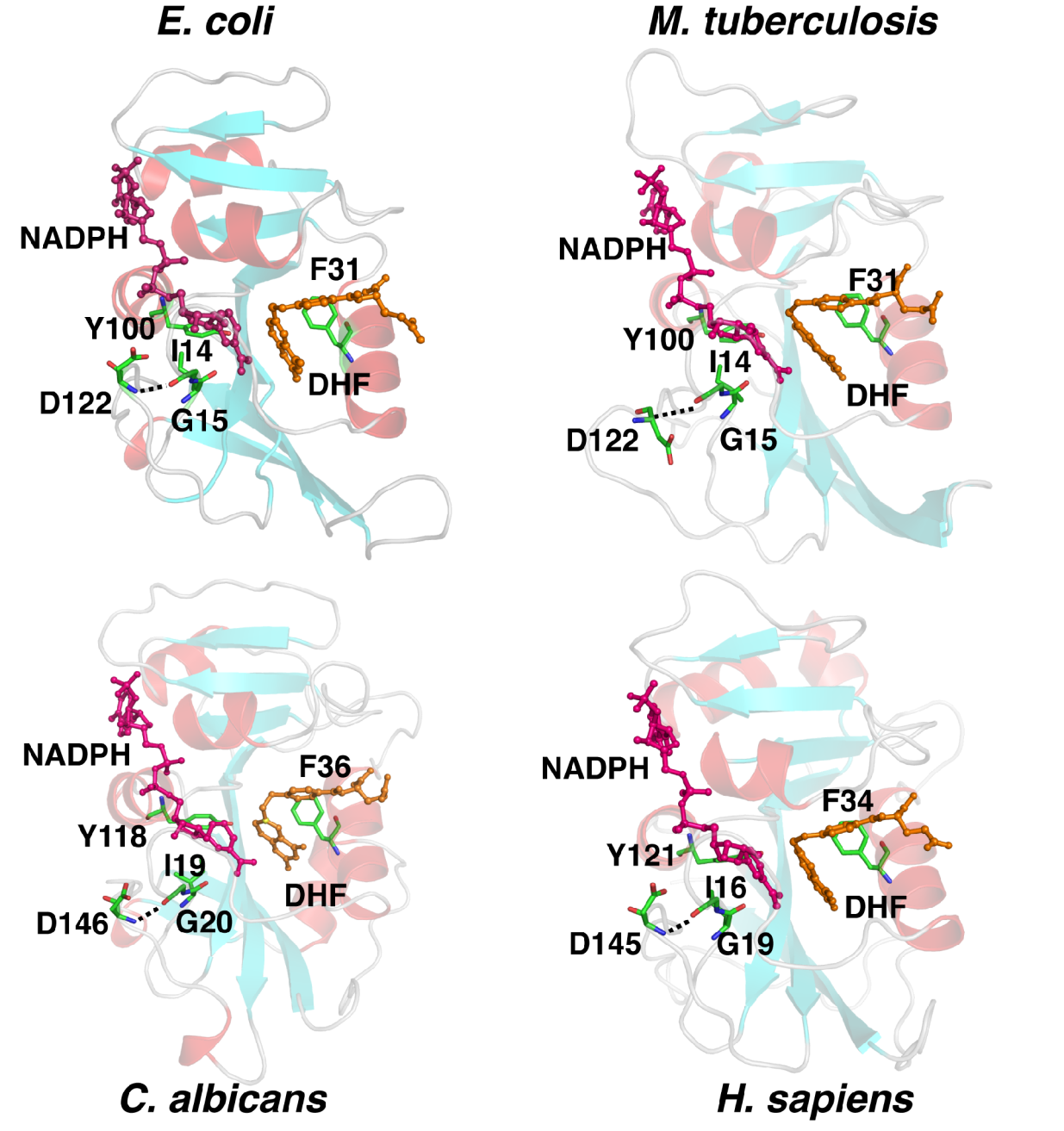

Supplement: Figure S7 — Details of the conserved network of coupled motions in enzyme DHFR. The flexible loops on the surface are connected to the active-site through conserved residues, hydrogen bonds, and hydrophobic interactions as listed in Table S4. (TIF) [file pbio.1001193.s007.tif]

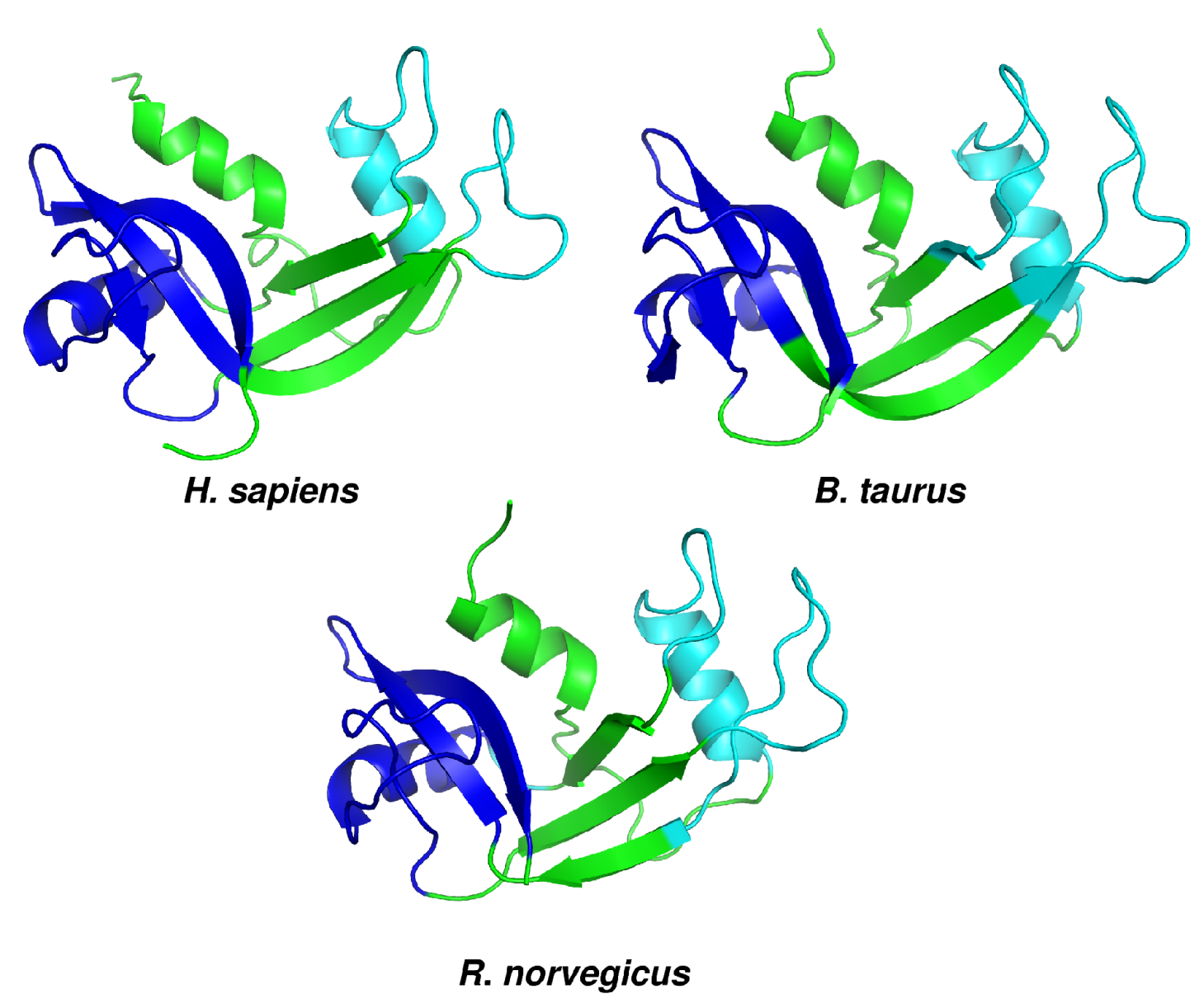

Supplement: Figure S8 — Dynamically coupled clusters in RNaseA. Three identical clusters were identified across the species. The three α-helices are clustered into three regions (blue, green, and cyan), indicating that the dynamics of these helices are quite different. The β-sheet is split into two distinct clusters (green and blue) depending on how these regions flank the substrate in the active site. The opposed movements of the β-sheet regions (see movies) and the motions of the flexible loop regions (cyan and blue regions) are a conserved dynamical feature of the RNaseA fold. (TIF) [file pbio.1001193.s008.tif]

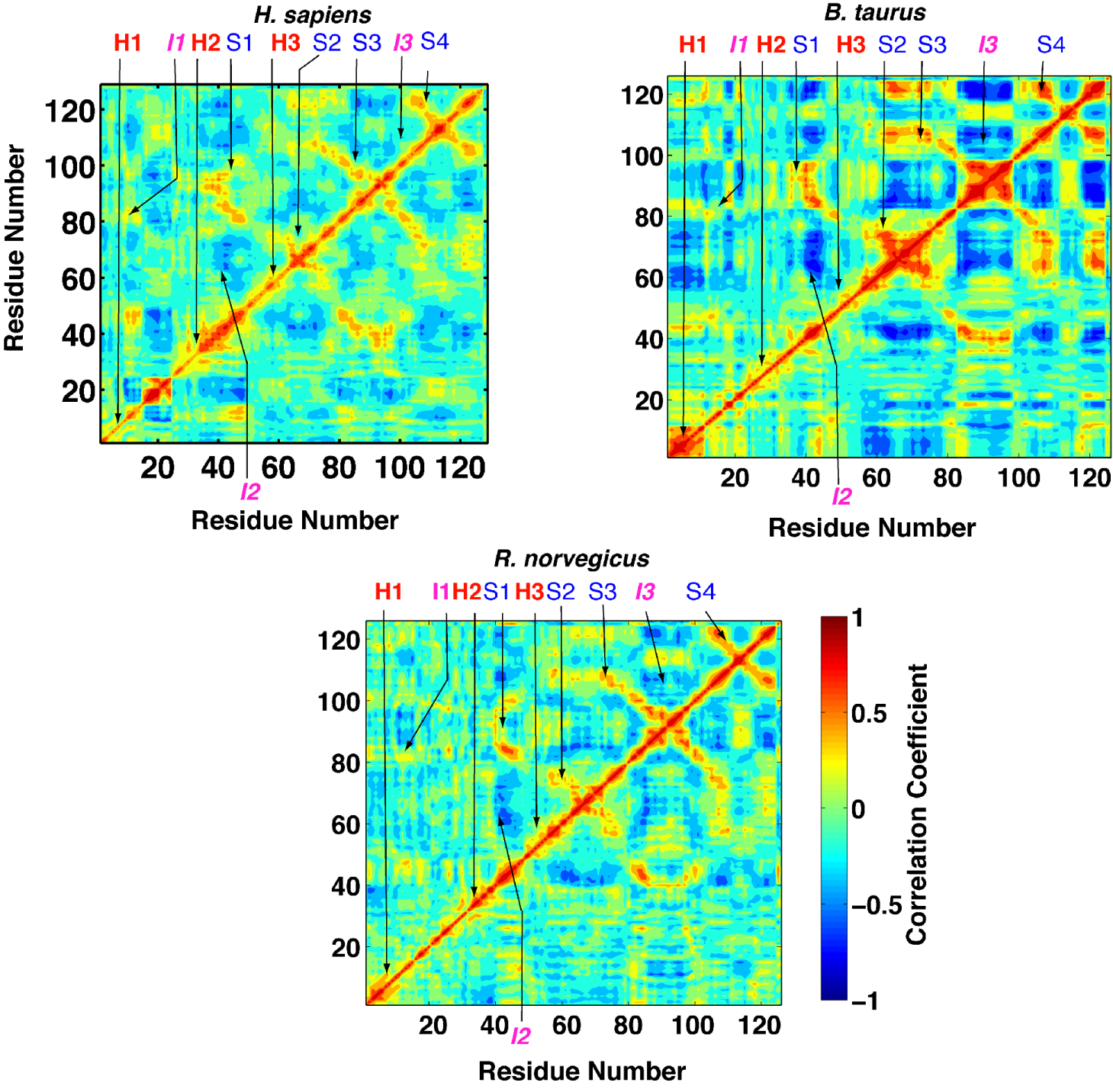

Supplement: Figure S9 — Cross-correlations in RNaseA: Regions H1–H3 and S1–S4 correspond to correlations observed from secondary structural elements (α1–α3, β1–β5), respectively. Regions I1–I2 correspond to distal correlations observed. The distal correlations observed from RNaseA are depicted in Table S5. (TIF) [file pbio.1001193.s009.tif]

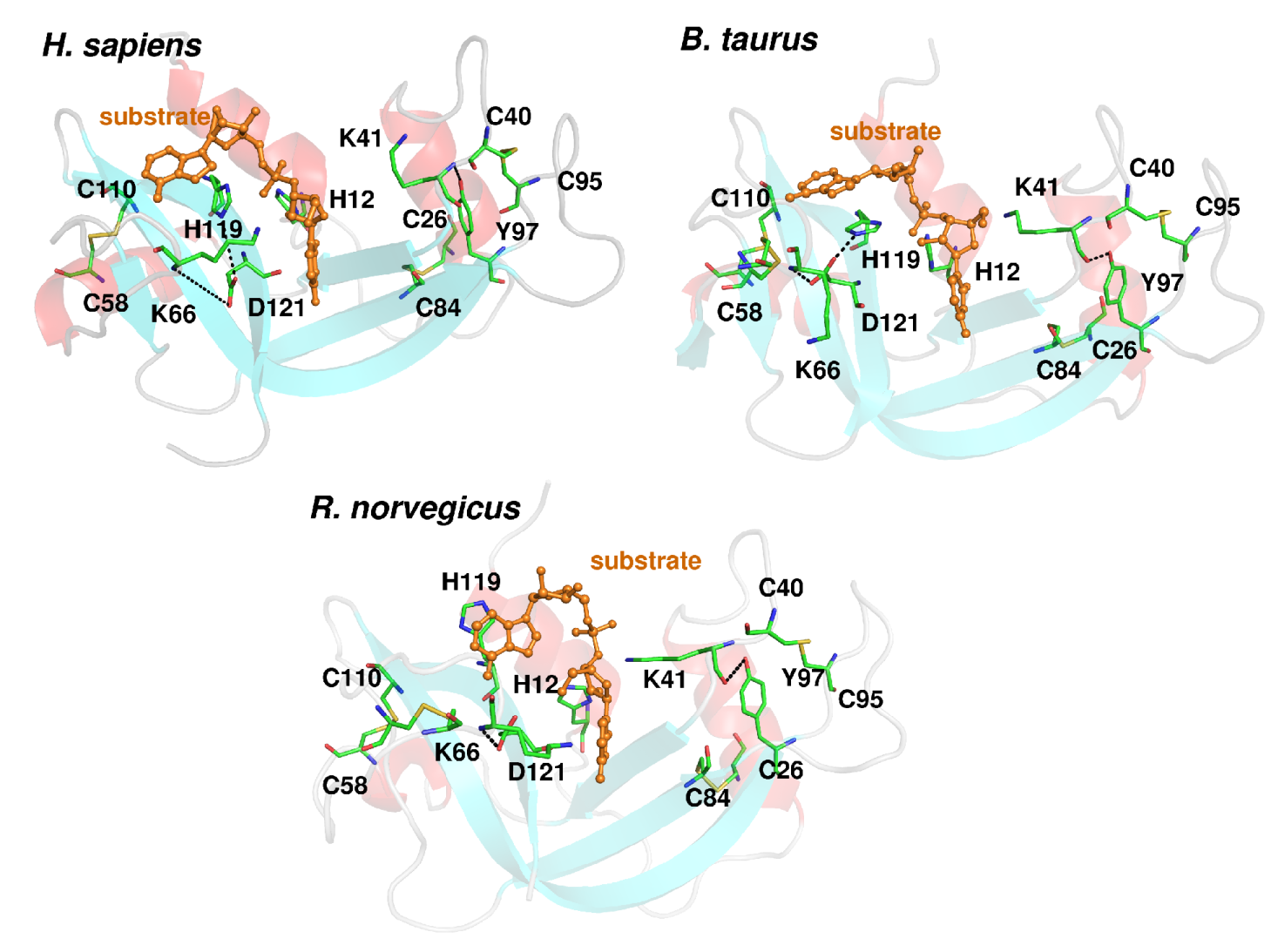

Supplement: Figure S10 — Details of conserved networks in RNaseA. The flexible loops on the surface are connected to the active-site through conserved residues and disulphide bonds as listed in Table S6. (TIF) [file pbio.1001193.s010.tif]

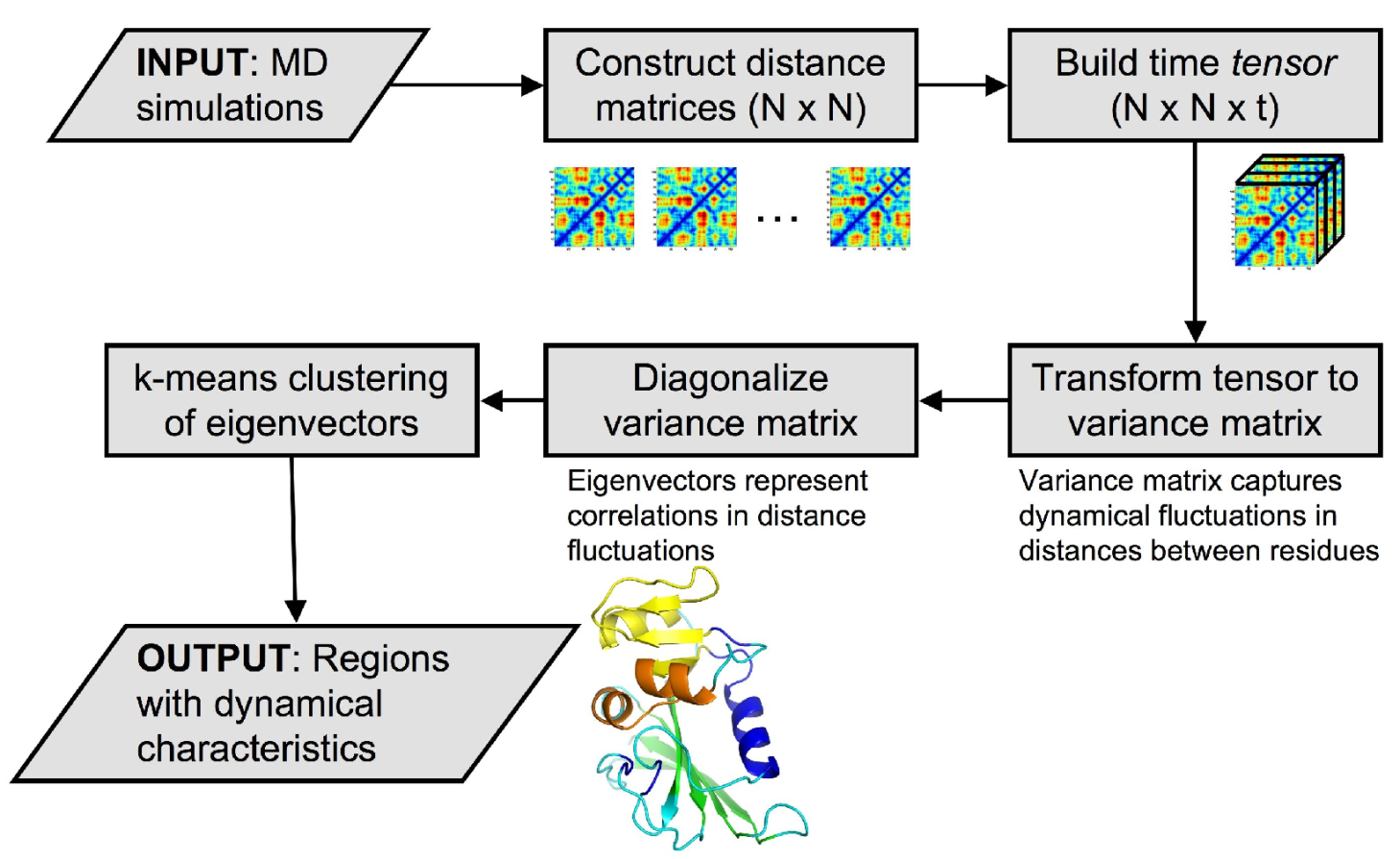

Supplement: Figure S11 — The dynamic cluster methodology for identification of protein regions exhibiting similar motions over the course of MD simulation(s). (TIF) [file pbio.1001193.s011.tif]

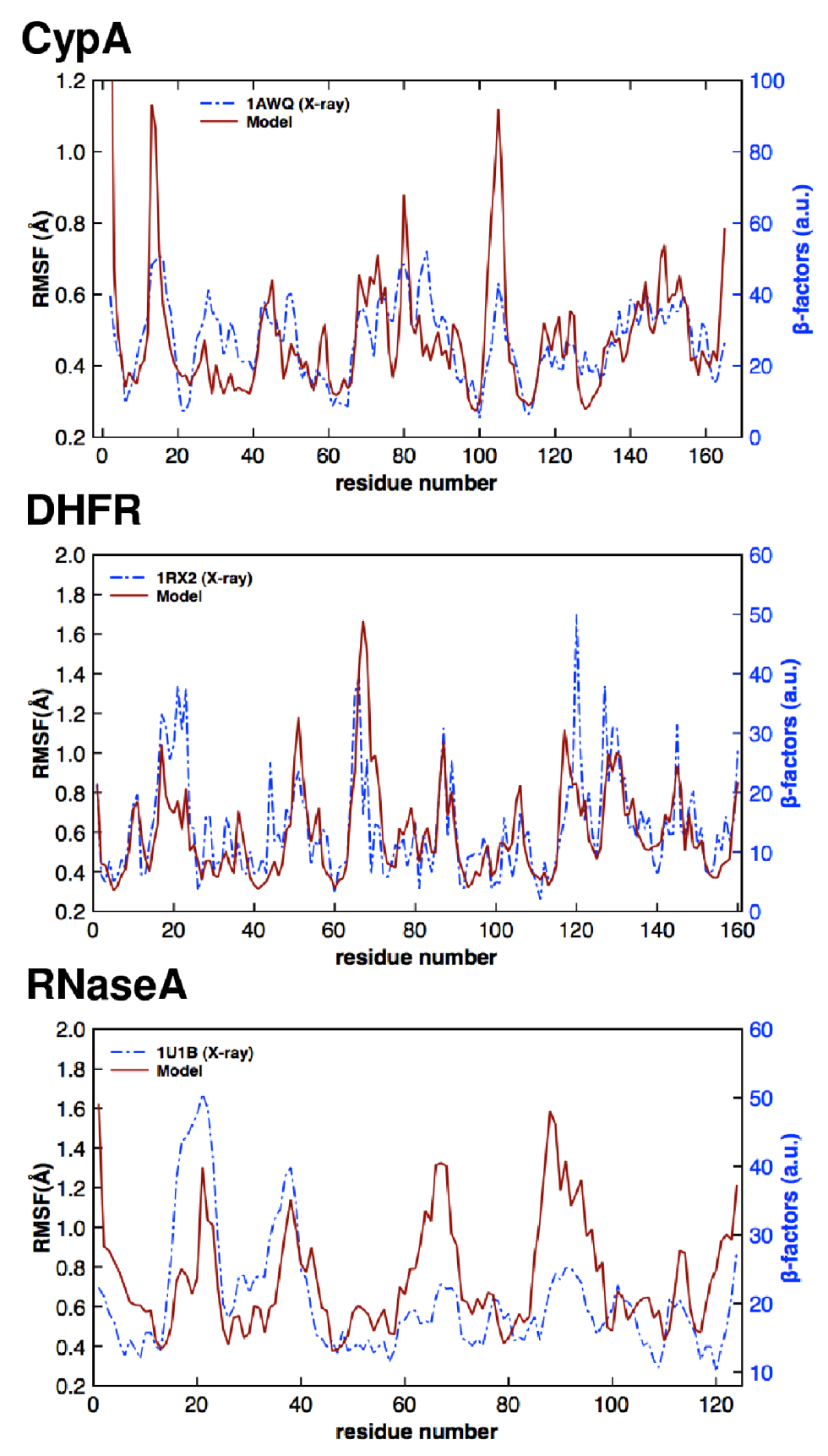

Supplement: Figure S12 — Validation of the enzyme flexibility. Comparison of the computationally obtained flexibility of the enzyme models (root mean square fluctuations) is compared with the temperature factors (β-factors) from the X-ray structures. The results show reproducible trends in high and low areas of enzyme flexibility. (TIF) [file pbio.1001193.s012.tif]
