# Supplementary material for: Evolutionarily Conserved Linkage between Enzyme Fold, Flexibility, and Catalysis
Source: PLoS Biol. 2011 Nov 8;9(11):e1001193. doi: 10.1371/journal.pbio.1001193 (PMC3210774; doi:10.1371/journal.pbio.1001193)
Supplement: Table S1 — CypA regions showing high correlations. (DOC) [file pbio.1001193.s022.doc]

Table S1. CypA regions showing high correlations.

| **Region** | ***H. sapiens*** | ***B. taurus*** | ***P. yeolii*** |
| --- | --- | --- | --- |
| I1 | 29–33 / 85–86 | 40–44 / 104–105 | 27–31 / 88–89 |
| I2 | 34–36 / 77–78 | 45–47 / 96–97 | 32–34 / 80–81 |
| I3 | 56–57 / 142–150 | 75–76 / 155–162 | 60–61 / 143–151 |
| I4 | 82–85 / 104–105 | 101–104 / 123–124 | 85–87 / 107–108 |
