# Supplementary material for: Evolutionarily Conserved Linkage between Enzyme Fold, Flexibility, and Catalysis
Source: PLoS Biol. 2011 Nov 8;9(11):e1001193. doi: 10.1371/journal.pbio.1001193 (PMC3210774; doi:10.1371/journal.pbio.1001193)
Supplement: Table S2 — Network interactions in PPIase fold. (DOC) [file pbio.1001193.s023.doc]

**Table S2. Network interactions in PPIase fold.**

| I | D13N-K155O | N35N_δ2_-G109O | I56N-G150O | A101N-Q111 | F83N-N108O |
| --- | --- | --- | --- | --- | --- |
| II | G21N-D164O | N43N_δ2_-G117O | V64N-D159O | A109N-Q119O | F91N-N116O |
| III | D16N-D167O | N38N_δ2_-G120O | V67N-N162O | A112N-Q122O | F94N-N119O |
| IV | G25N-L175O | N47N_δ2_-G129O | I76N-E170O | A121N-Q131O | F103N-N128O |
| V | N7N-I156O | N26N_δ2_-T95O | V44N-D149O | A86N-Q97O | I68N-A94O |
