# Supplementary material for: Evolutionarily Conserved Linkage between Enzyme Fold, Flexibility, and Catalysis
Source: PLoS Biol. 2011 Nov 8;9(11):e1001193. doi: 10.1371/journal.pbio.1001193 (PMC3210774; doi:10.1371/journal.pbio.1001193)
Supplement: Table S3 — DHFR regions showing high correlations. (DOC) [file pbio.1001193.s024.doc]

**Table S3. DHFR regions showing high correlations.**

| **Region** | ***E. coli*** | ***M. tuberculosis*** | ***C. albicans*** | ***H. sapiens*** |
| --- | --- | --- | --- | --- |
| I1 | 15–22 / 116–125 | 15–22 / 116–125 | 16–26 / 140–150 | 14–24 / 142–149 |
| I2 | 31–36 / 142–150 | 31–36 / 142–150 | 36–45 / 178–186 | 34–43 / 170–176 |
| I3 | 64–72 / 142–150 | 66–74 / 142–150 | 78–90 / 178–186 | 76–87 / 170–176 |
