# Supplementary material for: Evolutionarily Conserved Linkage between Enzyme Fold, Flexibility, and Catalysis
Source: PLoS Biol. 2011 Nov 8;9(11):e1001193. doi: 10.1371/journal.pbio.1001193 (PMC3210774; doi:10.1371/journal.pbio.1001193)
Supplement: Table S4 — Network interactions in DHFR fold. (DOC) [file pbio.1001193.s025.doc]

Table S4. Network interactions in DHFR fold.

| ***E. coli*** | D122N–G15O | I14–Y100 | Y100–NADPH | F31–DHF |
| --- | --- | --- | --- | --- |
| ***M. tuberculosis*** | D122N–G15O | I14–Y100 | Y100–NADPH | F31–DHF |
| ***C. albicans*** | D146N–G20O | I19–Y118 | Y118–NADPH | F36–DHF |
| ***H. sapiens*** | D145N–G19O | I16–Y121 | Y121–NADPH | F34–DHF |
