# Supplementary material for: Evolutionarily Conserved Linkage between Enzyme Fold, Flexibility, and Catalysis
Source: PLoS Biol. 2011 Nov 8;9(11):e1001193. doi: 10.1371/journal.pbio.1001193 (PMC3210774; doi:10.1371/journal.pbio.1001193)
Supplement: Table S5 — RNaseA regions showing high correlations. (DOC) [file pbio.1001193.s026.doc]

Table S5. RNase A regions showing high correlations.

| **Region** | ***H. Sapiens*** | ***B. taurus*** | ***R. norvegicus*** |
| --- | --- | --- | --- |
| I1 | 15–19/ 79–83 | 15–19/ 79–83 | 15–19/ 79–83 |
| I2 | 62–76/ 40–51 | 62–76/ 40–51 | 62–76/ 40–51 |
| I3 | 86–97/ 100–105 | 86–97/ 100–105 | 86–97/ 100–105 |
