# Supplementary material for: Evolutionarily Conserved Linkage between Enzyme Fold, Flexibility, and Catalysis
Source: PLoS Biol. 2011 Nov 8;9(11):e1001193. doi: 10.1371/journal.pbio.1001193 (PMC3210774; doi:10.1371/journal.pbio.1001193)
Supplement: Table S6 — Network interactions in RNaseA fold. (DOC) [file pbio.1001193.s027.doc]

Table S6. Network interactions in RNase A fold.

| ***H. sapiens*** | C26-C84 | C40-C95 | C58-C110 | H12 | Y97OH-K41O |
| --- | --- | --- | --- | --- | --- |
| ***B. taurus*** | C26-C84 | C40-C95 | C58-C110 | H12 | Y97OH-K41O |
| ***R. norvegicus*** | C26-C84 | C40-C95 | C58-C110 | H12 | Y97OH-K41O |
