# Supplementary material for: Evolutionarily Conserved Linkage between Enzyme Fold, Flexibility, and Catalysis
Source: PLoS Biol. 2011 Nov 8;9(11):e1001193. doi: 10.1371/journal.pbio.1001193 (PMC3210774; doi:10.1371/journal.pbio.1001193)
Supplement: Text S1 — Detailed results of Pin1 computational modeling, details of clustering methodology of protein regions based on conformational fluctuations, and details of the computational methodology used for calculating the dynamical cross-correlation maps are provided. (DOC) [file pbio.1001193.s028.doc]

Additional Supporting Information for article “Evolutionarily conserved linkage between enzyme fold, flexibility and catalysis”

*Arvind Ramanathan, Pratul K. Agarwal*

**Animation movies of the modes: CypA, DHFR and RNaseA**

The following movies are available with this document in MPEG format: Animation movies of the CypA (top 3 modes coupled to *cis/trans* isomerization reaction: Movies S1-S3); DHFR (top 3 modes coupled to the hydride transfer reaction: Movies S4-S6) and RNaseA (slowest 3 modes in the reactant–product ensemble: Movies S7-S9). Mode A, B, and C correspond the modes showing largest coupling, second largest coupling and third largest coupling to the reaction coordinate respectively. For RNaseA the motions of the substrate have been removed and Modes 1, 2 and 3 correspond to the lowest eigenvalue, second lowest eigenvalue and third lowest eigenvalue in QHA respectively. The flexible regions are color coded (similarly across species) based on their impact on the catalyzed reaction. See legends of Figures 1, 3 and 5 (main manuscript) for the coloring scheme used and other details.

**Consensus sequences**

The consensus sequences (CS1.xls) for CypA, DHFR and RNaseA are provided in Microsoft Excel format in Text S2.

**Pin1 Results**

Pin1 was modeled based on human Pin1 X-ray crystal structure (PDB code: 1PIN). Only the PPIase domain, residues 45–163, was used for model building. Model substrate pSer–Pro was modeled based on the position of the peptide Ala–Pro present in the X-ray structure. The enzyme–substrate complex was immersed in a box of explicit solvent. The rotation of the peptide bond catalyzed by Pin1 was modeled based on the protocol followed previously for the CypA simulations [25]. The Pin1 results are shown in Figure S4.

**Computational methodology details: Description of the clustering methodology**

A dynamic clustering methodology (dynamic tensor analysis) is a new data informatics approach to identify protein regions that exhibit similar dynamical characteristics observed over a period of time, such as the course of enzyme reaction [81]. In this clustering methodology, a series of protein conformations sampled by molecular dynamics (or multiple runs along the reaction coordinates) are analyzed for dynamical correlation in Cartesian space with time representing an extra dimension for tensor analysis (see Figure S11). Following diagonalization, K-means clustering algorithm is used to obtain optimal number of clusters for separating the flexible protein regions exhibiting similar characteristics over the course of enzyme reaction, allowing identification of correlated regions in a set of slow protein conformational fluctuations. This method offers an advantage over cross-correlation matrix by separating regions with dissimilar dynamics and over quasi-harmonic analysis as it summarizes information pertaining to the correlated regions in a set of slow protein conformational fluctuations.

**Computational methodology: dynamical cross-correlation**

Cross-correlation coefficient for the displacement of any two atoms *i* and *j* is defined as:

$\mathbf{C}_{ij}=\frac{\langle\Delta\mathbf{r}_{i}\cdot\Delta\mathbf{r}_{j}\rangle}{\left(\langle\Delta\mathbf{r}_{i}^{2}\rangle\langle\Delta\mathbf{r}_{j}^{2}\rangle\right)^{1/2}}$,

where Δ**r***_i_* is the displacement of the *i*th atom from its mean position, and quantity in áñ denotes an average over the set of conformations collected during the reaction pathway (in case of RNaseA only the reactant and product states). **C***_ij_* matrix is visualized as dynamical cross-correlation maps depicted in Figures S2, S6, and S9 (for CypA, DHFR and RNase A respectively. Note, the results correspond to mass-weighted cross-correlations between residues. All conformations collected during MD simulations and the reaction pathways were used for this analysis. The dynamical cross-correlations allow identification of important residues pairs that show coupled motions over the reaction pathway [25].
